# Loss of Christianson Syndrome Na^+^/H^+^ Exchanger 6 (NHE6) Causes Abnormal Endosome Maturation and Trafficking Underlying Lysosome Dysfunction in Neurons

**DOI:** 10.1523/JNEUROSCI.1244-20.2021

**Published:** 2021-11-03

**Authors:** Matthew F. Pescosolido, Qing Ouyang, Judy S. Liu, Eric M. Morrow

**Affiliations:** ^1^Department of Molecular Biology, Cell Biology and Biochemistry, Laboratories for Molecular Medicine, Brown University, 70 Ship Street, Providence, RI 02912; ^2^Center for Translational Neuroscience, Carney Institute for Brain Science and Brown Institute for Translational Science, Brown University, 70 Ship Street, Providence, RI 02912; ^3^Department of Neurology, Rhode Island Hospital, 593 Eddy Street, Providence, RI 02903

**Keywords:** Christianson syndrome, endosome, exosome, lysosome, neurodegeneration, NHE6

## Abstract

Loss-of-function mutations in endosomal Na^+^/H^+^ exchanger 6 (NHE6) cause the X-linked neurologic disorder Christianson syndrome. Patients exhibit symptoms associated with both neurodevelopmental and neurodegenerative abnormalities. While loss of NHE6 has been shown to overacidify the endosome lumen, and is associated with endolysosome neuropathology, NHE6-mediated mechanisms in endosome trafficking and lysosome function have been understudied. Here, we show that NHE6-null mouse neurons demonstrate worsening lysosome function with time in culture, likely as a result of defective endosome trafficking. NHE6-null neurons exhibit overall reduced lysosomal proteolysis despite overacidification of the endosome and lysosome lumen. Akin to Nhx1 mutants in *Saccharomyces cerevisiae*, we observe decreased endosome-lysosome fusion in NHE6-null neurons. Also, we find premature activation of pH-dependent cathepsin D (CatD) in endosomes. While active CatD is increased in endosomes, CatD activation and CatD protein levels are reduced in the lysosome. Protein levels of another mannose 6-phosphate receptor (M6PR)-dependent enzyme, β-N-acetylglucosaminidase, were also decreased in lysosomes of NHE6-null neurons. M6PRs accumulate in late endosomes, suggesting defective M6PR recycling and retromer function in NHE6-null neurons. Finally, coincident with decreased endosome-lysosome fusion, using total internal reflection fluorescence, we also find a prominent increase in fusion between endosomal multivesicular bodies and the plasma membrane, indicating enhanced exosome secretion from NHE6-null neurons. In summary, in addition to overacidification of endosomes and lysosomes, loss of NHE6 leads to defects in endosome maturation and trafficking, including enhanced exosome release, contributing to lysosome deficiency and potentially leading to neurodegenerative disease.

**SIGNIFICANCE STATEMENT** Loss-of-function mutations in the endosomal Na^+^/H^+^ exchanger 6 (NHE6) cause Christianson syndrome, an X-linked neurologic disorder. Loss of NHE6 has been shown to overacidify endosomes; however, endosome trafficking mechanisms have been understudied, and the mechanisms leading to neurodegeneration are largely unknown. In NHE6-null mouse neurons *in vitro*, we find worsening lysosome function with days in culture. Notably, pH-dependent lysosome enzymes, such as cathepsin D, have reduced activity in lysosomes yet increased, precocious activity in endosomes in NHE6-null neurons. Further, endosomes show reduced fusion to lysosomes, and increased fusion to the plasma membrane with increased exosome release. This study identifies new mechanisms involving defective endosome maturation and trafficking that impair lysosome function in Christianson syndrome, likely contributing to neurodegeneration.

## Introduction

The nervous system is vulnerable to endolysosome dysfunction. Lysosomal storage disorders, which are caused by mutations in lysosome-associated genes, provide primary evidence linking endolysosomal dysfunction with neurologic disease (Platt et al., 2012; Boustany, 2013). Additional genetic studies have further implicated endolysosomal genes in more common neurodegenerative diseases, such as Alzheimer's disease and Parkinson's disease (Fraldi et al., 2016; Sharma et al., 2018; Winckler et al., 2018; Lie and Nixon, 2019).

The process of endosome maturation involves the transition of endosomes in the earlier stages of the endocytic pathway to the degradative route, thereby ensuring proper trafficking of cargo assigned for degradation to the lysosome (Huotari and Helenius, 2011; Scott et al., 2014). Progressive acidification of the endolysosomal lumen from early to late stages of the endocytic pathway is a key feature of endosome maturation, and regulated by a number of membrane-based ion channels, pumps, and transporters (Mellman et al., 1986; Casey et al., 2010; Huotari and Helenius, 2011). Endosomal Na^+^/H^+^ exchangers (NHEs) (i.e., NHE6 and NHE9) represent a class of cation exchangers that are thought to alkalinize intralumenal pH by exchanging luminal H^+^ for cytosolic cations (e.g., Na^+^ or K^+^). NHE6 protein has been shown to be particularly abundant on early and recycling endosomes (>80%) (Brett et al., 2002; Ohgaki et al., 2010; Ouyang et al., 2013), and is also on late endosomes (∼50%) at least in neurons (Ouyang et al., 2013).

Exemplifying the importance of endosomal NHEs to human brain function, loss-of-function mutations in X-linked *NHE6* cause the neurologic disorder Christianson syndrome (CS) (Gilfillan et al., 2008; Pescosolido et al., 2014). Affected males present with intellectual disability, epilepsy, ataxia, and postnatal microcephaly (Pescosolido et al., 2014). Neurodegenerative features identified in CS patients include cerebellar degeneration and widespread neuronal loss and gliosis, potentially involving tau deposition (Garbern et al., 2010; Pescosolido et al., 2014); however, the cellular mechanisms mediating neurodegenerative pathology in CS remain unclear. Ouyang et al. (2013) found that loss of NHE6 in neurons led to an overacidification of the endosomal lumen, consistent with a critical role for NHE6 in the regulation of luminal pH during endosome maturation. The functions of NHE6 in endosome maturation and trafficking are less well understood. Abnormalities in these primary endosome processes may secondarily lead to defects in lysosome function. Neuropathological features seen in lysosome disorders have been previously reported in the CS mouse model (Stromme et al., 2011; Sikora et al., 2016); however, the molecular mechanisms underlying potential lysosome deficiency in CS are unknown.

In this study, we investigate mechanisms of endolysosome dysfunction in NHE6-null neurons. We show here that loss of NHE6 in primary neurons *in vitro* causes worsening lysosome function with days in cultures, and that this lysosome deficiency is associated with defects in endosome maturation and trafficking. NHE6-null neurons exhibit overall reduced lysosomal proteolysis, despite overacidification of the endosome and lysosome lumen. Akin to Nhx1 mutants in *Saccharomyces cerevisiae*, the yeast homolog of NHE6 (Karim and Brett, 2018), we observe decreased endosome-lysosome fusion in NHE6-null neurons. We find precocious activation of the pH-dependent lysosome enzyme cathepsin D (CatD) in endosomes, with evidence of reduced CatD trafficked to lysosomes. While loss of NHE6 inhibits endosome-lysosome fusion, we find greater fusion between late endosomes/multivesicular bodies (MVBs) with the plasma membrane (PM), leading to enhanced exosome release. Overall, these novel disease mechanisms in CS, involving defective endosome maturation likely causing lysosome deficiency, place CS within the context of a growing category of neurodegenerative disorders with endolysosome dysfunction.

## Materials and Methods

### Materials

Materials included protease inhibitor (Roche, 05892970001), phosSTOP (Roche, 04906837001), PMSF (Sigma, 93482), Fluoromount-G (Southern Biotechnology), Lipofectamine 2000 (Invitrogen, catalog #11668019), 22 mm poly-D-lysine-coated coverslips (Neuvitro, GG-22-1.5-PDL), and black CellCarrier-96 Ultra microplates (PerkinElmer). The following compounds were used: 4-methyl-umbellyferyl-*N*-acetyl-β-D-glucosaminide (Millipore Sigma, 474502), bafilomycin A1 (Sigma, B1793), DMSO (Sigma, D8418), ionomycin (Sigma, I9657), NH_4_Cl (Sigma, A9434), and U18666A (Sigma, U3633). The following items were purchased through Thermo Fisher Scientific: AlexaFluor-546-transferrin (T23364), AlexaFluor-594-BSA (A13101), AlexaFluor-594-dextran (D22913), AlexaFluor-647-dextran (D22914), B27 (17504044), BODIPY FL-pepstatin A (P12271), DQ-BSA (Green, D12050), FITC-transferrin (T2871), GlutaMAX (35050061), Oregon Green (OG) 488-dextran (D7171), ProLong Gold antifade mountant with DAPI (P36931), and tetramethylrhodamine (TMR)-dextran (D1817).

### Antibodies

The following antibodies were used for western blot: actin (Sigma, A3853, Ms, 1:1000), CatD (R&D Systems, AF1029, Gt, 1:1000), CD63 (Abcam, EPR21151-ab217345, Rb, 1:1000), ci-mannose 6-phosphate receptor (M6PR) (Cell Signaling Technology, 14364S, Rb, 1:500), GAPDH (Sigma, G8795, Ms, 1:40 000), LAMP1 (DSHB, 1D4B, Rt, 1:1000), RAB5 (Cell Signaling Technology, 3547, Rb, 1:1000), and RAB7 (Sigma, R8779, Ms, 1:1000). The following antibodies were used for immunocytochemistry experiments: ci-M6PR (Abcam, 2G11-ab2733, Ms, 1:1000), Hoechst-33342 (Thermo Fisher Scientific, H1399, 1:1600), LAMP1 (Abcam, ab24170, Rb, 1:500 for BODIPY-pepstatin A, 1:1000 for M6PR), lysobisphosphatidic acid (LBPA, Echelon Biosciences, Ms, 1:100), MAP2 (Abcam, ab5392, Ch, 1:5000), RAB5 (Cell Signaling Technology, 3547, Rb, 1:500), RAB7 (Abcam, ab137029, Rb, 1:100 for BODIPY-pepstatin A, 1:500 for M6PR and 3D imaging), and TGN46 (Abcam, ab16059, Rb, 1:1000). The following secondary antibodies were used at a 1:800 dilution: anti-mouse AlexaFluor-488, anti-chicken AlexaFluor-594, anti-rabbit AlexaFluor-594, anti-mouse AlexaFluor-647, anti-rabbit AlexaFluor-647, and anti-rat AlexaFluor-647.

### Animals

All animal care and use were performed in accordance with National Institutes of Health guidelines and were approved under a protocol by the Brown University Institutional Animal Care and Use Committee.

### Dissociated hippocampal cultures

Dissociated hippocampal neurons were derived from mouse pups at P0-P1 as previously described (Ouyang et al., 2013). Cells were typically seeded at densities of either 3.0 × 10^5^/ml (immunocytochemistry) or 3.5 × 10^5^/ml (immunoblot and enzyme assays) unless otherwise specified. All treatments were added to Neurobasal media supplemented with B27 (2%), and Glutamax (1%) unless otherwise specified. Cultured neurons were transfected at DIV 13 with Lipofectamine 2000 (Invitrogen).

### Western blot

Western blots were analyzed by the Odyssey Clx Infrared Imaging System (LI-COR) and Odyssey software version 5.2.5. Primary hippocampal neurons were washed with cold 1× PBS and lysed with RIPA buffer supplemented with 1% protease inhibitor on ice. Cultures were then scraped, put on ice for 1 h, and centrifuged for 10 min at 13,000 RPM at 4°C. Mouse hippocampal tissue was harvested at 8 weeks, lysed with tissue lysis buffer (RIPA buffer supplemented with 1% protease inhibitor, 1% phosSTOP and 1% PMSF), homogenized, and put on ice for 1 h. Brain tissue samples were then centrifuged for 15 min at 13,200 RPM at 4°C. Protein quantity was quantified for all samples using a BCA protein assay. Primary hippocampal cultures (5 or 10 µg) or hippocampal tissue (30 µg) were resolved on NuPAGE 4%-12% SDS-polyacrylamide gels (Invitrogen). All proteins of interest were normalized to loading control proteins (e.g., actin or GAPDH). Western blot images have been cropped for presentation.

### Lysosomal enzyme assays

Enzyme activity was measured in 8-week-old mouse brain tissue (e.g., hippocampus, cerebellum, and cortex) and 14 DIV hippocampal neuronal cultures. β-N-Acetylglucosaminidase (β-NAG) activity was measured using the substrate 4-nitrophenyl *N*-acetyl-β-D-glucosaminide (NP-GlcNAc) according to the kit protocol (Sigma, CS0780). Acid phosphatase activity was measured using the substrate 4-nitrophenyl phosphate according to the kit protocol (Sigma, CS0740). Hippocampal primary culture (10 µg) or mouse brain tissue (20 µg) was incubated in triplicate for 10 min at 37°C. Absorbance was measured at 405 nm on a Cytation3 microplate reader (BioTek) using Gen5 software version 2.07.

### Lysosome-enriched fractionation

Lysosomes from the cortex and hippocampus of 4-month-old male *Nhe6^-/Y^* and WT littermate mice were enriched using the lysosome isolation kit (LYSISO1, Sigma). The modified protocol from Bagh et al. (2017) was used. Briefly, brain tissue was homogenized in 4 volumes of 1× extraction buffer with protease inhibitor and spun for 10 min at 1000 × *g* at 4°C. The supernatant was then centrifuged for 20 min at 20,000 × *g* at 4°C. The crude lysosome fraction pellet was resuspended in 1× extraction buffer and added to the 19% Optiprep gradient. The following Optiprep gradients were layered: 27%, 22.5%, 19% (including crude lysosome fraction), 16%, 12%, and 8%. Samples were ultracentrifuged (Optima MAX-XP, Beckman Coulter) for 4 h at 150,000 × *g* at 4°C. Five fractions were collected at the junction of each gradient. Fractions 2 (12%-16%) and 3 (16%-19%), which had the highest LAMP1 protein levels, were combined and used for analysis. The same amount of protein was loaded for Western blot (2.5 µg). Proteins of interest were normalized to LAMP1.

### Confocal microscopy

Confocal *z*-stack for the following experiments were acquired using an LSM 800 (Carl Zeiss) microscope: (1) DQ-BSA, (2) AF-594 BSA, and (3) BODIPY-pepstatin A (see Fig. 2) experiments. Images were collected using an oil-immersion 63× objective with 1024 × 1024 pixel resolution. To ensure an unbiased selection, all neurons were selected using the DIC channel. For colocalization experiments (e.g., BODIPY-pepstatin A and M6PR), *z*-stack images were acquired using an Olympus FV3000 microscope. Images were collected using an oil-immersion 63× objective with 512 × 512 pixel resolution. pH and endosome-lysosome fusion experiments were imaged using the Opera Phenix High-Content Screening System (PerkinElmer). Single-plane (pH) or *z*-stack (endosome-lysosome fusion) images were collected with a water-immersion 63× objective. For each experiment, laser settings were the same across all time points. For live imaging, cells were placed in a humidity chamber and maintained at 37°C with 5% CO_2_.

### DQ-BSA degradation

Mouse hippocampal neuronal cultures were analyzed at DIV 3, 5, and 14. Cells were treated with 20 µg/ml of DQ-BSA at 37°C. Cells were incubated with DQ-BSA for 30 min, washed twice with 1× PBS, and chased for 1.5 h with supplemented Neurobasal media. Following DQ-BSA treatment, neurons were briefly rinsed with 1× PBS and fixed with 4% PFA for 10 min at room temperature. Cells were the washed with 1× PBS 3 times for 5 min each, with the first and second washes containing Hoechst. Slides were mounted with Fluoromount-G.

### BSA uptake

Mouse hippocampal neuronal cultures were analyzed at DIV 3, 5, and 14. Cells were treated with 20 µg/ml of BSA conjugated with AlexaFluor-594 (BSA-AF594) at 37°C. Cells were incubated with BSA-AF594 for 30 min, briefly rinsed twice with 1× PBS, and fixed with 4% PFA for 10 min at room temperature. Cells were then washed with 1× PBS 3 times for 5 min each, with the first and second washes containing Hoechst. Slides were mounted with Fluoromount-G.

### Active CatD labeling using BODIPY FL-pepstatin A

Mouse hippocampal neuronal cultures were analyzed at DIV 3, 5, and 14. Cells were treated with 1 µg/ml of BODIPY FL-pepstatin A for 1 h at 37°C. Following BODIPY FL-pepstatin A treatment, neurons were briefly rinsed twice with 1× PBS and fixed with 4% PFA for 10 min at room temperature. Cells were then washed 3 times with 1× PBS for 5 min each, with the first and second washes containing Hoechst. Slides were mounted with Fluoromount-G.

### Active CatD colocalization with endolysosomal markers

Mouse hippocampal neuronal cultures were analyzed at DIV 5 and 14 with the same BODIPY FL-pepstatin A treatment as the prior experiment. Following treatment, cells were washed 3 times with warm 1× PBS, fixed/permeabilized with 4% PFA in 1× PBS for 30 min at room temperature, and washed 3 times with 1× PBS for 5 min per wash. For dextran experiments, cells were then mounted with Fluoromount-G. For non-dextran experiments, cells were then blocked with 10% goat serum in 1× PBS and 0.1% Triton X-100 or 0.01% saponin (LBPA) for 1 h at room temperature. Cells were then incubated with primary antibodies overnight in 1× PBS and 0.1% Triton X-100 or 0.01% saponin (LBPA) at 4°C. The next day, cells were washed twice with 1× PBS, incubated with secondary antibodies in 1× PBS and 0.1% Triton X-100 or 0.01% saponin (LBPA) for 1 h at room temperature, washed once with 1× PBS, and mounted with Fluoromount-G. For dextran experiments, cells were incubated with 5 mg/ml dextran (1:25) for 3 h at 37°C at DIV 4 or 13. Cells were washed twice with 1× PBS and chased overnight (∼16 h) with Neurobasal media.

### Endosome and lysosome pH

Mouse hippocampal neuronal cultures were analyzed at DIV 8. For each animal, 3 or 4 wells were seeded in CellCarrier-96 Ultra microplates (PerkinElmer) for replication purposes. Lysosome pH was measured using a protocol adapted from Johnson et al. (2016). At DIV 7, cells were incubated with 0.1 mg/ml each of OG 488-dextran (OG-dextran) and TMR-dextran (TMR-dextran) for 2 h at 37°C. They were washed 3 times with 1× PBS and then chased overnight in supplemented Neurobasal media. Before live imaging, cells were incubated with Hoechst-33342 (1:1600) in supplemented Neurobasal media-minus phenol red for 10 min to label cell nuclei. They were then washed once with 1× PBS and imaged with supplemented Neurobasal media-minus phenol red. The fluorescence ratio was converted to absolute pH using a pH calibration curve. The calibration curve was generated by imaging pH standards (e.g., 3.5, 4.5, and 5.5) in a calibration solution (125 mm KCl, 25 mm NaCl, 10 µm monensin, 25 mm MES, and adjusted to a final pH using 1N NaOH or 1N HCl). For bafilomycin A1 experiments, 100 nm was added with Hoechst-33342 in supplemented Neurobasal media-minus phenol red for 10 min. Cells were then imaged in supplemented Neurobasal media-minus phenol red with 100 nm of bafilomycin A1. Endosome pH was measured in primary hippocampal neurons at DIV 5 as previously described (Ouyang et al., 2019).

### Endosome-lysosome fusion

Cells were seeded on CellCarrier-96 Ultra microplates (PerkinElmer). Mouse hippocampal neuronal cultures were analyzed at DIV 5. At DIV 4, cells were incubated with 0.25 mg/ml of TMR-dextran for 2 h at 37°C. Cells were then washed twice with 1× PBS and chased overnight with supplemented Neurobasal media. At DIV 5, cells were incubated with 0.5 mg/ml AlexaFluor-647-dextran and Hoechst-33342 (1:5000) for 10 min at 37°C, washed twice with 1× PBS, and imaged immediately with supplemented Neurobasal media-minus phenol red. For bafilomycin A experiments, cells were treated with 100 nm bafilomycin A1 while incubating with AlexaFluor-647-dextran and Hoechst-33342. Live-cell imaging was performed using the Opera Phenix High-Content Screening System in supplemented Neurobasal media-minus phenol red. *z*-stack images were taken every 20 min over the span of 2 h (i.e., 7 time points).

### Total internal reflection fluorescence (TIRF) microscopy

Exosome secretion events were visualized by ring-TIRF using the DeltaVision OMX SR imaging system (GE). Mouse hippocampal neuronal cultures were analyzed at 14 DIV. Cells were plated on 22 mm poly-D-lysine-coated coverslips (Neuvitro) at 3.5 × 10^5^/ml density. At 13 DIV, cells were cotransfected with CD63-pHluorin and empty-mCherry plasmids using Lipofectamine 2000. Before imaging, neurons were transferred to Tyrode's solution (124 mm NaCl, 3 mm KCl, 2 mm CaCl_2_, 1 mm MgCl_2_, 10 mm HEPES, pH 7.4, 5 mm D-glucose). Images were collected on a 63× TIRF objective at 1024 × 1024 resolution with oil 1.522. To visualize transfected cell location, a widefield, single-plane image was collected before TIRF imaging. Laser settings were identical across all experiments. Environmental settings were constant for O_2_ (20%), CO_2_ (5%), and humidity (50%). Five-minute videos were collected per cell at 2 Hz (i.e., every 500 ms). Cultured neurons were treated with bafilomycin A1 (100 nm for 2 h) and U18666A (1.5 μg/ml for 16 h) (Strauss et al., 2010).

### Image acquisition and analysis

For lysosome pH experiments, 20 images were collected per well for each sample. Data were collected from at least three independent experiments using at least 4 animals per genotype. Image analysis was performed using Harmony software (version 4.9, PerkinElmer). The DAPI channel was used to define the nucleus and cell soma regions using the “Find Nuclei” and “Find Cytoplasm” building blocks, respectively. Cell count was calculated from identified nuclei. Live cells were then distinguished using a minimum nuclear area threshold. Dextran-labeled lysosomes were identified using the “Find Spots” building block. Spots (i.e., lysosomes) that met inclusion criteria for fluorescence intensity and size (15-140 pixels^2^) were included for analysis. Fluorescence intensity for both OG- and TMR-dextran channels was measured per spot. Spots within the “Find Nuclei” building block were classified as soma, whereas all other spots were classified as processes. To determine lysosome pH, the ratio of OG-dextran fluorescence intensity to TMR-dextran fluorescence intensity was calculated. A calibration curve was generated by plotting the values of the OG-dextran/TMR-dextran ratio against the pH values obtained from the pH standards for each experiment. Fluorescent intensity values collected from experimental samples were converted to pH values using the calibration curve formula. Endosome pH analysis was performed as previously described (Ouyang et al., 2019).

For active CatD labeling experiments, 20 images were selected for each sample. Data were collected from at least four independent experiments using at least four different mouse litters. Sample file names were randomized to ensure unbiased analysis. BODIPY-pepstatin A images were analyzed using ImageJ software (National Institutes of Health). Before analysis, the same background subtraction was applied to all images (i.e., rolling ball radius 50 pixels). Puncta quantification was performed using the “Analyze Particles” function. The same image settings were applied to all images: subtract background (30), threshold (70). Mean fluorescence intensity (MFI) was calculated by outlining individual neurons in the DIC channel using the Freehand tool and measuring green channel fluorescence. A background measurement was collected using the Oval tool to draw an area size between 20 and 30. MFI was calculated as: mean intensity – background.

For active CatD colocalization experiments, 10 images were selected for each sample. Data were collected from at least three independent experiments using at least three different mouse litters. A single-plane image was selected, cropped, and channels were separated in ImageJ. The following thresholds were applied: MaxEntropy (BODIPY-pepstatin A), Intermodes (LAMP1, RAB7, LBPA, RAB5), and RenyiEntropy (dextran). Colocalization was calculated using the Manders' coefficient in JACoP (Bolte and Cordelieres, 2006). Analysis settings include the following: confocal, wavelength A = 488, wavelength B = 647 (LAMP1, RAB7, LBPA, RAB5) or 568 (dextran), NA = 1.4, refractive index = 1.518. Calibration settings were selected using “Get calib. From ImgA,” which reported the following values: *xy* calibration = 96.57 and *z* calibration = 1000.

M6PR colocalization experiment parameters were nearly identical to active CatD colocalization experiments (see above). The following thresholds were applied: MaxEntropy (M6PR, TGN46, RAB7), Moments (LAMP1), and Intermodes (RAB5). Analysis settings include the following: confocal, wavelength A = 488 (M6PR), wavelength B = 647 (all other markers).

For endosome-lysosome fusion experiments, *z*-stacks from the same 6 ROIs were collected for each sample across all time points. Data were collected from five independent experiments using 7 animals per genotype. Image analysis was performed using Harmony software (version 4.9) similar to lysosome pH experiments. The DAPI channel was used to define the nucleus using the “Find Nuclei” building block. Cell count was calculated from identified nuclei. Live cells were then distinguished using a minimum nuclear area threshold. Dextran-labeled vesicles were identified using the “Find Spots” building block. Spots (i.e., TMR-dextran and AlexaFluor-647-dextran) that met inclusion criteria for fluorescence intensity were included for analysis. Percent of fusion events (i.e., AlexaFluor-647-dextran + TMR-dextran spots/Total AlexaFluor-647-dextran spots) was analyzed using the “Find Population” building block with AlexaFluor-647-dextran as population 1 and TMR-dextran as population 2. Fusion event data are expressed as % fold change to time point 0 for each animal.

3D-reconstruction analysis was performed using Imaris 5.1 software (Bitplane). *z*-stack confocal images were cropped, and volume was reconstructed: surface and “MIP” (i.e., maximum intensity projection). File names were randomized to ensure unbiased analysis. Puncta were manually thresholded by adjusting: (1) background subtraction, (2) absolute intensity, and (3) split touching objects. To segment the nucleus, the DAPI marker was manually traced throughout the entire *z*-stack. The distance between different markers was calculated using the “Distance Transformation” module, with “Outside SurfaceObject” selected.

### Lysosome exocytosis experiments

Media was collected from mouse primary hippocampal cultures at DIV 14. Approximately 8 × 10^5^ cells were added to a well of a 6-well plate. For each sample, primary cultures were seeded in duplicate to allow for treatment comparisons. Neurons were treated with either ionomycin (10 µm) or DMSO for 10 min at 37°C. Media was then collected and centrifuged for 10 min at 13,200 at 4°C to pellet any cellular debris.

β-Hexosaminidase (β-Hex) activity was measured using a protocol adapted from Laulagnier et al. (2011) and using the Tyrode's solution previously mentioned. Following media collection, cells were washed once with cold 1× PBS and lysed with 500 μl of cell lysis buffer (RIPA buffer + 1% protease inhibitor). Cells were put on ice for 15 min, spun for 10 min at 13,200 RPM at 4°C, and the supernatant was collected. For media and cell lysate samples, 50 μl was added to a 96-well in triplicate. In each well, the following were added: 5 μl of lysine, 16 μl of reaction mixture (1 mm 4-methyl-umbellyferyl-*N*-acetyl-β-D-glucosaminide in 11.2 mm citrate, 17.6 mm Na_2_HPO_4_, pH 4.5), and 45 μl of 1× PBS. Samples were incubated for 30 min at 37°C, followed by the addition of 100 μl of stop reaction (2 m Na_2_CO_3_, 1.1 m glycine, pH 10.2). Fluorescence was measured at Ex/Em = 365/450 nm on a Cytation3 microplate reader (BioTek) using Gen5 software version 2.07. β-Hex activity was calculated by averaging each sample and subtracting the control sample average (i.e., Tyrode's solution without any primary neurons). Released activity was calculated using the following formula: (media – control media)/(cell lysis – cell lysis buffer) × 100.

CatD activity was measured using the synthetic substrate GKPILFFRLK(Dnp)-D-R-NH_2_ according to the kit protocol (Abcam, ab65302). Culture media was aspirated at 14 DIV and replaced with 1 ml of Tyrode's solution (124 mm NaCl, 3 mm KCl, 2 mm CaCl_2_, 1 mm MgCl_2_, 10 mm HEPES, pH 7.4, 5 mm D-glucose) before treatment. A total of 50 μl of sample media was added per well in duplicate and incubated for 1.5 h at 37°C. Fluorescence was measured at Ex/Em = 328/460 nm on a Cytation3 microplate reader (BioTek) using Gen5 software version 2.07. Enzyme activity was calculated by subtracting the media-only average from each sample average.

LDH was measured and calculated according to the protocol (Sigma, MAK066). For each sample, 50 μl of media was added to a 96-well plate in duplicate. Absorbance was measured at 450 nm on a Cytation3 microplate reader (BioTek) using Gen5 software version 2.07.

### Statistical analysis

Data are presented as mean ± SEM. *n* represents the number of biological replicates for each experiment. For microscopy experiments, the *n* typically represents the number of cells, although it may also represent the number of animals (e.g., pH and endosome-lysosome fusion experiments) analyzed. Most statistical analyses were performed using GraphPad Prism version 7. Normality was assessed using the D'Agostino and Pearson omnibus normality test. For experiments with a low sample size (*n* < 7) where D'Agostino and Pearson omnibus normality test was unable to be tested, Gaussian distribution was assumed. Unless otherwise specified, two group comparisons were analyzed by unpaired, two-tailed Student's *t* test (data normally distributed) or Mann–Whitney *U* test (data not normally distributed). For data not normally distributed with distribution shapes between the 2 groups, a log10 transformation was applied and a normality test was performed on the transformed data. Effect size was calculated using Cohen's *d* (sample size *n* > 20), Hedges' *g* (samples size *n* < 20), or Glass's Δ (if SD significantly different). Linear mixed model analysis was performed using SPSS version 25. Endosome-lysosome fusion experiments were analyzed using a linear mixed model, with % endosome-lysosome fusion as the dependent variable. Genotype and time points were factors, whereas litter was entered as a covariate. Intercept, genotype, time, litter, and time × genotype were modeled as fixed effects, whereas animal was modeled as a random effect. Time points (e.g., 20, 40, 60, 80, 100, and 120) were calculated as % endosome-lysosome fusion normalized to time point 0. The time × genotype interaction for % endosome-lysosome fusion was the primary outcome variable. Analysis of exosome secretion following various treatments was analyzed using Kruskal–Wallis test with Dunn's test for multiple comparisons. An ordinal logistic regression was performed using Stata SE (release 15). The number of MVB-PM fusion/exosome release events (i.e., counts) were considered ordered categorical variables. Lysosomal exocytosis experiments were performed with two-way ANOVA followed by Tukey's multiple comparisons test when necessary.

## Results

### Loss of NHE6 leads to reduced lysosomal protease function

**Figure 1.**
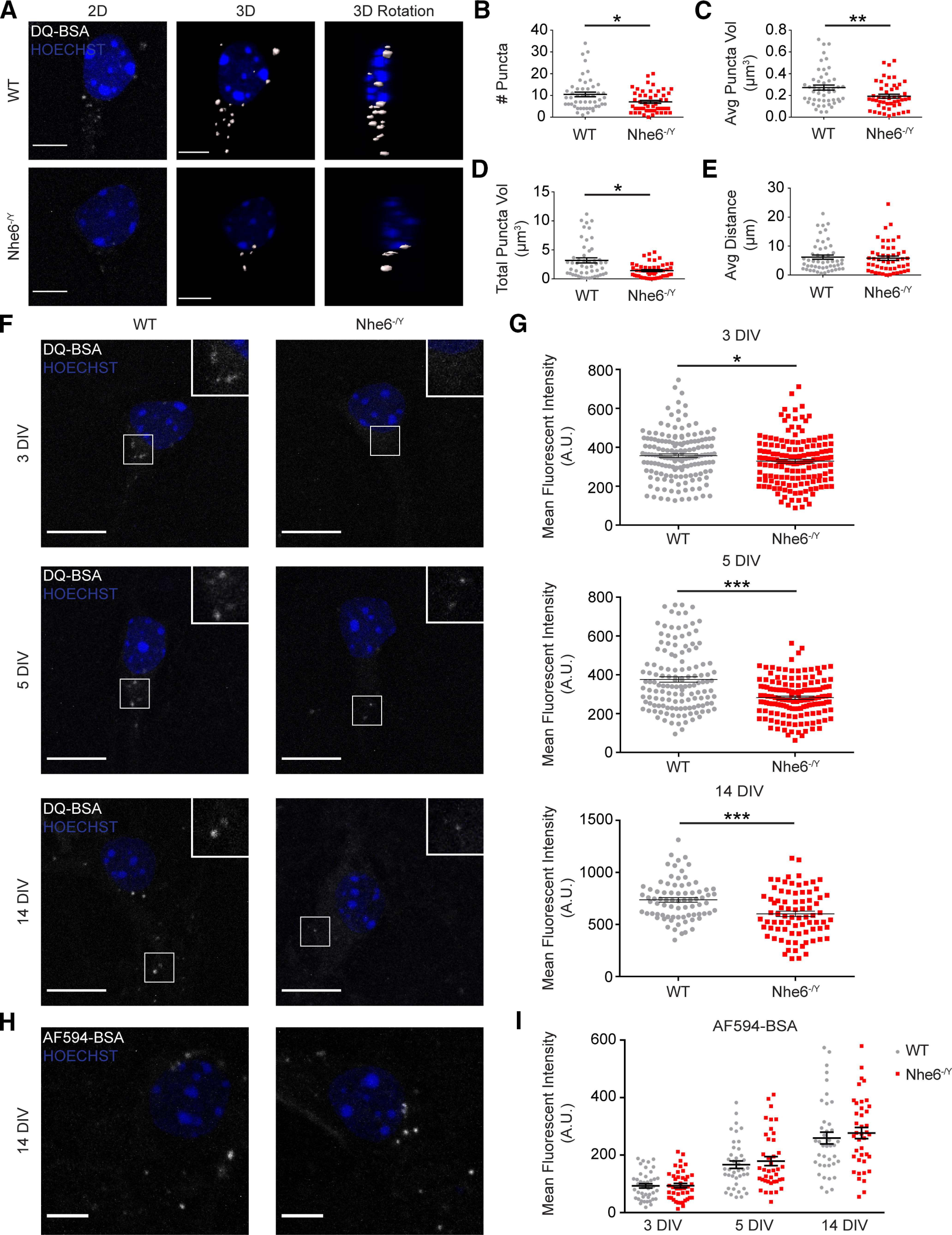
Decreased lysosomal proteolysis in NHE6-null neurons *in vitro*. ***A***, Puncta analysis by fluorescence microscopy and 3D images of WT and *Nhe6^-/Y^* male primary hippocampal neurons following DQ-BSA treatment at DIV 5. Images are denoted as: 2D (i.e., DQ-BSA fluorescent microscopy maximum intensity projection images), 3D (i.e., 3D reconstruction of DQ-BSA puncta), and 3D Rotation (i.e., 3D image rotated 90° along the *x* axis). ***B–E***, Quantification of 3D-reconstructed DQ-BSA puncta. ***B***, Number of DQ-BSA puncta per cell (WT *n* = 50 cells from 5 mice, *Nhe6^-/Y^ n* = 50 cells from 5 mice, 4 litters, *p* = 0.02, Glass's Δ = 0.46). ***C***, Average DQ-BSA puncta volume per cell (WT *n* = 50 cells from 5 mice, *Nhe6^-/Y^ n* = 49 cells from 5 mice, 4 litters, *p* = 0.002, Cohen's *d* = 0.54). ***D***, Total summed DQ-BSA puncta volume per cell (WT *n* = 50 cells from 5 mice, *Nhe6^-/Y^ n* = 49 cells from 5 mice, 4 litters, *p* = 0.01, Glass's Δ = 0.58). ***E***, Average distance of DQ-BSA puncta from nucleus (WT *n* = 50 cells from 5 mice, *Nhe6^-/Y^ n* = 49 cells from 5 mice, 4 litters). ***F***, Confocal microscopy images of male WT and *Nhe6-null* littermate primary hippocampal neurons following DQ-BSA treatment at DIV 3, 5, and 14. ***G***, Quantification of MFI at 3 DIV (WT *n* = 160 cells, *Nhe6^-/Y^ n* = 156 cells, 5 mice per genotype, 4 litters, *p* = 0.04, Cohen's *d* = 0.23), 5 DIV (WT *n* = 138 cells, *Nhe6^-/Y^ n* = 143 cells, 5 mice per genotype, 4 litters, *p* < 0.0001, Glass's Δ = 0.58), or 14 DIV (WT *n* = 78 cells, *Nhe6^-/Y^ n* = 80 cells, 4 mice per genotype, 4 litters, *p* < 0.0001, Cohen's *d* = 0.64). Some primary neurons at 5 DIV were analyzed in both 3D reconstruction and MFI data. ***H***, Fluorescence microscopy images of WT and *Nhe6^-/Y^* male primary hippocampal neurons following BSA-AF594 treatment at 14 DIV. ***I***, Quantification of MFI at 3 DIV (WT *n* = 42 cells, *Nhe6^-/Y^ n* = 43 cells, 5 mice per genotype, 5 litters), 5 DIV (WT *n* = 40 cells, *Nhe6^-/Y^ n* = 40 cells, 5 mice per genotype, 5 litters), or 14 DIV (WT *n* = 40 cells, *Nhe6^-/Y^ n* = 40 cells, 5 mice per genotype, 5 litters). Nuclei are marked in blue by Hoechst. Scale bars: ***A***, ***H***, 5 µm; ***F***, 10 µm. Data are mean ± SEM. Unpaired two-tailed Student's *t* test (***G***, 3 and 14 DIV; ***I***, 3 and 14 DIV) or Mann–Whitney test (***B-E***,***G***, 5 DIV; ***I***, 5 DIV).

To examine the relationship between loss of NHE6 and lysosome functioning, we investigated the degradative capacity of primary hippocampal neurons *in vitro*. We treated NHE6-null and WT male neurons with DQ-BSA (i.e., BSA conjugated with fluorophores that emit only when degraded) to measure proteolysis of endocytosed material (Vazquez and Colombo, 2009). We first examined DQ-BSA puncta features using 3D reconstruction of primary hippocampal neurons at 5 DIV (Fig. 1*A*). This time point was chosen as some of the earliest overacidified endosomes findings in NHE6-null neurons were observed at 5 DIV (Ouyang et al., 2013). NHE6-null neurons had significantly fewer DQ-BSA puncta (Fig. 1*B*), smaller average individual punctum size (Fig. 1*C*), and less total summed puncta volume per cell (Fig. 1*D*) compared with WT neurons. There were no differences in DQ-BSA distribution across the cell, as measured by distance from the nucleus (Fig. 1*E*). To further investigate lysosome degradation, we measured the mean fluorescence intensity (MFI) of DQ-BSA treated neurons across a range of *in vitro* time points that reflect key neuronal processes, such as axonal outgrowth (3 DIV), dendritic outgrowth (5 DIV), and synaptic maturation (14 DIV) (Dotti et al., 1988; Craig and Banker, 1994). NHE6-null neurons displayed significantly decreased proteolytic activity across all three time points compared with WT littermate controls, with worsening dysfunction at older time points. Effect size by Cohen's *d* was 0.23 compared with 0.64 at 3 and 14 DIV, respectively (Fig. 1*F*,*G*). Importantly, this decrease in degradative signal was not because of differences in BSA internalization (Fig. 1*H*,*I*). These results indicate that loss of NHE6 leads to worsening lysosomal protease activity with time *in vitro* relative to control.

### NHE6-null neurons have reduced overall CatD activity

Hydrolases perform the degradative function of lysosomes, and their activity is dependent on the highly acidic lysosomal lumen (Braulke and Bonifacino, 2009). Lysosomal enzyme dysfunction represents a shared mechanism across many neurologic disorders leading to the accumulation of macromolecules in cells (Boustany, 2013; Lloyd-Evans and Haslett, 2016; Mazzulli et al., 2016). Based on our observations of deficient lysosomal protease function in NHE6-null neurons, we next examined the function of specific lysosome enzymes. To examine lysosome enzyme functioning, we analyzed the aspartic protease CatD whose dysfunction has been reported across multiple neurodegenerative disorders (Vidoni et al., 2016).

**Figure 2.**
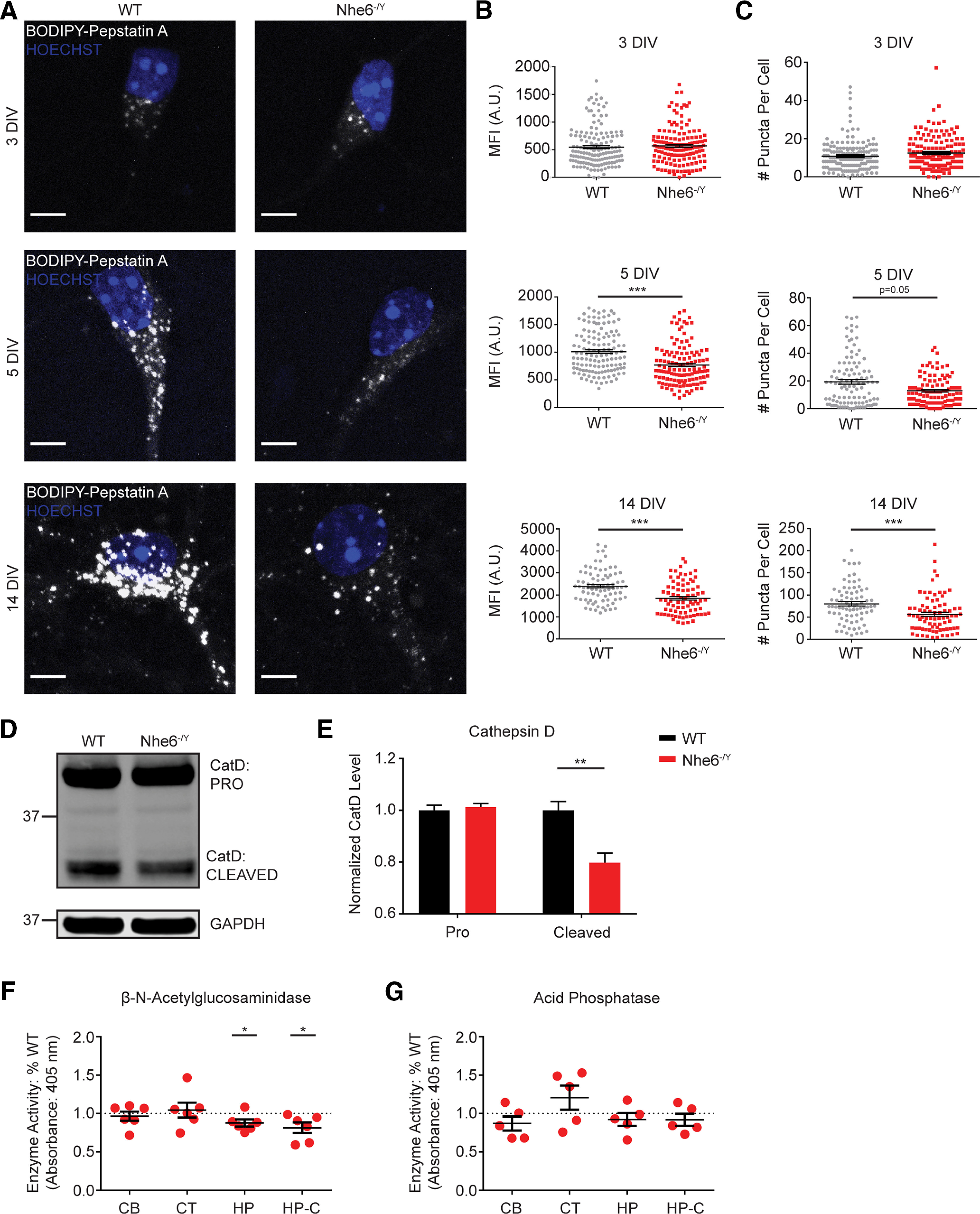
Loss of NHE6 impairs lysosome enzyme function *in vitro* and *ex vivo*. ***A***, Confocal microscopy images of mature CatD using BODIPY-pepstatin A in WT and *Nhe6^-/Y^* male mouse primary hippocampal neurons at DIV 3, 5, and 14. ***B***, Quantification of MFI per cell at 3 DIV (WT *n* = 153 cells, *Nhe6^-/Y^ n* = 148 cells, 5 mice per genotype, 5 litters), 5 DIV (WT *n* = 130 cells, *Nhe6^-/Y^ n* = 129 cells, 5 mice per genotype, 3 litters, *p* < 0.0001, Cohen's *d* = 0.66), and 14 DIV (WT *n* = 80 cells, *Nhe6^-/Y^ n* = 80 cells, 4 mice per genotype, 4 litters, *p* < 0.0001, Cohen's *d* = 0.76). ***C***, Quantification of number (#) of puncta per cell at 3 DIV (WT *n* = 161 cells, *Nhe6^-/Y^ n* = 148 cells, 5 mice per genotype, 5 litters), 5 DIV (WT *n* = 107 cells, *Nhe6^-/Y^ n* = 105 cells, 4 mice per genotype, 3 litters, *p* = 0.05, Glass's Δ = 0.37), and 14 DIV (WT *n* = 78 cells, *Nhe6^-/Y^ n* = 78 cells from, 4 mice per genotype, 4 litters, *p* < 0.0001, Cohen's *d* = 0.56). ***D***, ***E***, CatD western blot (***D***) and quantification (***E***) in WT and *Nhe6^-/Y^* male littermate mice, acutely dissected hippocampal tissue at 8 weeks old (WT *n* = 11 animals, *Nhe6^-/Y^ n* = 6 animals, 6 litters, cleaved CatD *p* = 0.003, Hedges' *g* = 1.77). ***F***, β-NAG and (***G***) acid phosphatase enzyme activity in *Nhe6^-/Y^* male littermate mice, acutely dissected brain tissue (CB, Cerebellum; CT, cortex; HP, hippocampus; *p* = 0.047, Hedges' *g* = 0.78) at 8 weeks old as well as primary hippocampal neurons at 14 DIV relative to male WT littermates (HP-C, Hippocampal culture, *p* = 0.044, Hedges' *g* = 1.42). The sample sizes are as follows: β-NAG brain tissue (WT *n* = 9 animals, *Nhe6^-/Y^ n* = 6 animals, 6 litters), β-NAG hippocampal culture (WT *n* = 6 animals, *Nhe6^-/Y^ n* = 6 animals, 6 litters), acid phosphatase brain tissue (WT *n* = 7 animals, *Nhe6^-/Y^ n* = 5 animals, 5 litters), and acid phosphatase hippocampal culture (WT *n* = 5 animals, *Nhe6^-/Y^ n* = 5 animals, 5 litters). Values are expressed as the percentage of *Nhe6^-/Y^* activity relative to its WT male littermate activity. Nuclei are marked in blue by Hoechst. Scale bars, 5 µm. Data are mean ± SEM. A.U., Arbitrary units; CB, cerebellum; CT, cortex; HP, hippocampus; HP-C, hippocampal cultures. One-sample Student's *t* test with a hypothetical mean = 1 (***F***,***G***), unpaired two-tailed Student's *t* test (***E***), or Mann–Whitney test (***B***,***C***).

To measure active CatD, we treated male *Nhe6^-/Y^* and WT littermate primary hippocampal neurons with BODIPY FL pepstatin A. For this probe, the CatD-specific inhibitor pepstatin A has been conjugated with a pH-insensitive fluorophore (Chen et al., 2000). NHE6-null neurons demonstrated significantly reduced active CatD activity at DIV 5 and 14 (Fig. 2*A*). Specifically, NHE6-null neurons had significantly decreased signal intensity (Fig. 2*B*) and fewer puncta (Fig. 2*C*), both worsening with days in culture. There were no differences in puncta size (data not shown). We investigated whether these CatD findings extended to mouse brain tissue. Therefore, we quantified active CatD protein levels biochemically in male *Nhe6^-/Y^* and WT littermate hippocampus at 8 weeks of age. Cleaved-CatD (i.e., the enzymatically active form) was significantly decreased in *Nhe6^-/Y^* mice (large effect size, Hedges' *g* = 1.77) (Fig. 2*D*,*E*).

To determine whether other lysosomal enzymes are affected, we measured the activity of β-NAG and acid phosphatase in NHE6 mouse brain tissue (e.g., cerebellum, cortex, and hippocampus) from acutely dissected brains at 8 weeks of age, as well as from primary hippocampal neurons at 14 DIV. β-NAG activity was significantly reduced in male NHE6-null hippocampal tissue as well as primary hippocampal cultures compared with male WT littermates [large effect size, Hedges' *g* = 0.78 (tissue) and Hedges' *g* = 1.42 (cultures)] (Fig. 2*F*). No differences were found in the cortex or cerebellum. There were no differences in acid phosphatase activity across all brain tissue regions and primary hippocampal neurons at 14 DIV (Fig. 2*G*). These findings may reflect differences in lysosomal enzyme trafficking routes. Many newly synthesized lysosomal enzymes are transported from the trans-Golgi network (TGN) to the endocytic pathway by binding to M6PRs, including CatD and β-NAG (von Figura and Hasilik, 1986; Ghosh et al., 2003). There is evidence to support the notion that acid phosphatase is trafficked via a distinct, M6PR-independent pathway (Braun et al., 1989; Peters et al., 1990). Together, these results indicate that loss of NHE6 leads to deficits *ex vivo* and *in vitro* in CatD and β-NAG function, with both enzymes being trafficked in a M6PR-dependent manner.

### Reduced lysosome intraluminal pH in NHE6-null neurons

**Figure 3.**
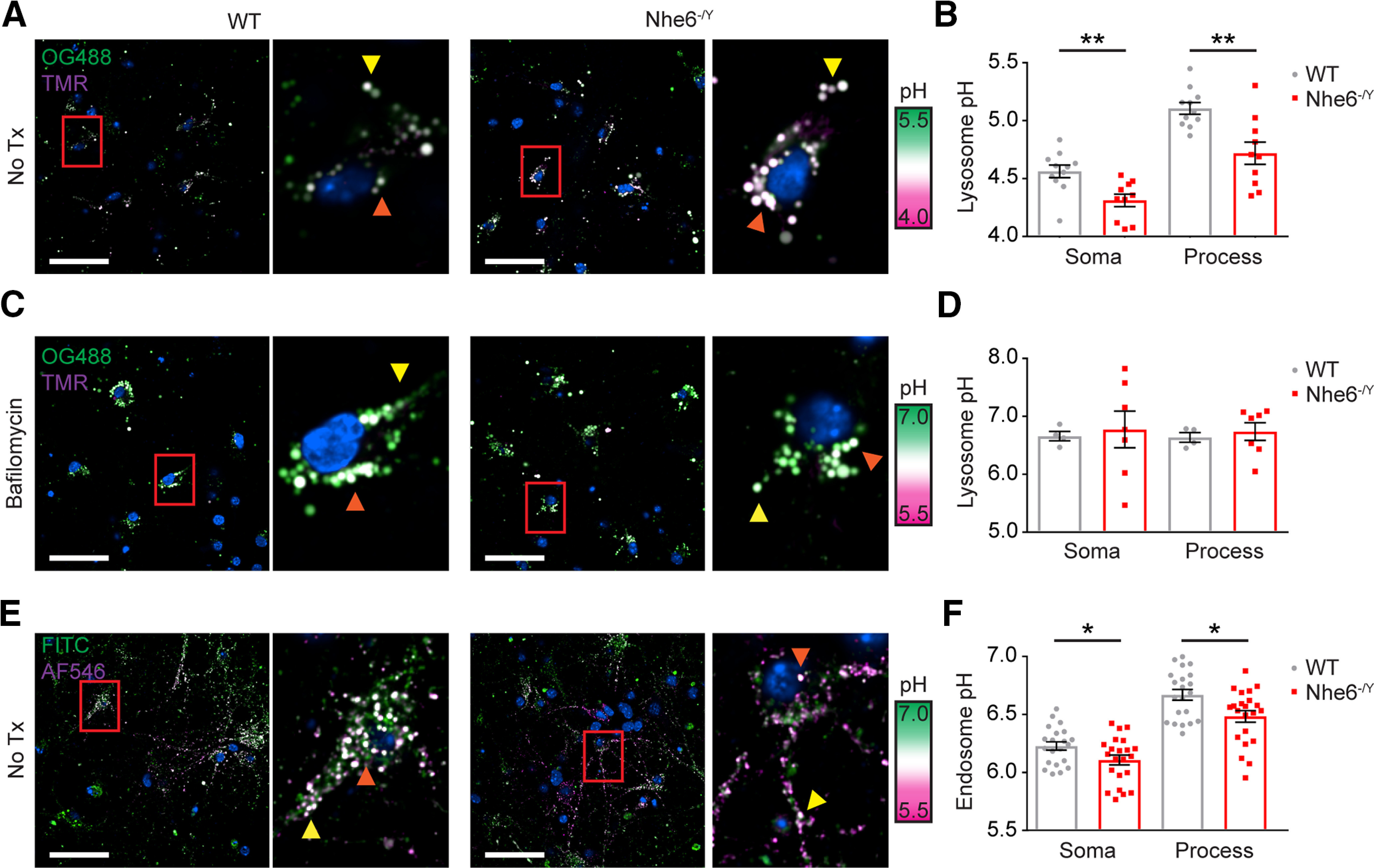
Intraluminal lysosome pH more acidic in NHE6-null neurons. ***A***, Single-plane confocal microscopy images of lysosome pH loaded with fluorescent dextrans (i.e., pH-sensitive OG 488-dextran and pH-insensitive TMR-dextran) in male WT and NHE6-null mouse primary hippocampal neurons at DIV 8. ***B***, Quantification of intraluminal lysosome pH in soma and processes (WT *n* = 11 animals, *Nhe6^-/Y^ n* = 10 animals, 5 litters; soma: *p* = 0.002, Hedges' *g* = 1.43; processes: *p* = 0.002, Hedges' *g* = 1.60). ***C***, Single-plane confocal microscopy images of lysosome pH loaded with fluorescent dextrans (i.e., pH-sensitive OG 488-dextran and pH-insensitive TMR-dextran) following bafilomycin A1 treatment (100 nm) in male WT and NHE6-null mouse primary hippocampal neurons at DIV 8. ***D***, Quantification of intraluminal lysosome pH in soma and processes following bafilomycin A1 treatment (WT *n* = 4 animals, *Nhe6^-/Y^ n* = 7 animals, 3 litters). ***E***, Single-plane confocal microscopy images of endosome pH loaded with fluorescent transferrin (i.e., pH-sensitive FITC-transferrin and pH-insensitive AlexaFluor-546-transferrin) in male WT and NHE6-null mouse primary hippocampal neurons at DIV 5. ***F***, Quantification of luminal endosome pH in soma and processes (WT *n* = 21, *Nhe6^-/Y^ n* = 22, 13 litters; soma: *p* = 0.04, Cohen's *d* = 0.66; processes: *p* = 0.01, Cohen's *d* = 0.83). Orange arrows indicate soma. Yellow arrowheads denote processes. Scale bars, 50 µm. Data are mean ± SEM. Unpaired two-tailed Student's *t* test (***B***, processes, ***F***) with Welch's correction (***D***, soma) or Mann–Whitney test (***B***, soma).

Given loss of NHE6 leads to hyperacidification of the endosome lumen *in vitro* (Ouyang et al., 2013), we sought to determine whether intraluminal pH in lysosomes was also affected. We adapted a ratiometric fluorescence microscopy protocol using dextran from Johnson et al. (2016) to measure the luminal pH of lysosomes. Primary hippocampal neurons were treated with both pH-sensitive (i.e., OG 488) and pH-insensitive (i.e., TMR) dextran and chased overnight to allow for trafficking to lysosomes (Fig. 3*A*). The fluorescence ratio was converted to absolute pH using a pH calibration curve. Using a high-content imaging system, we found that NHE6-null neurons had significantly lower intralysosomal pH compared with WT male neurons (Fig. 3*B*). Specifically, both the soma and processes contained more acidic lysosomes in NHE6-null neurons. Primary hippocampal neurons were treated with bafilomycin A1, as a positive control, to alkalinize the luminal pH of lysosomes (Fig. 3*C*). As expected, lysosomal pH increased and there were no significant differences between NHE6-null and WT neurons (Fig. 3*D*). To further verify our prior results on intra-endosomal pH in NHE6-null cells, but now using our high-content imaging system, we examined transferrin-positive early/recycling endosome pH using fluorescent ratio imaging (Fig. 3*E*). NHE6-null neurons had significantly lower endosome pH in both the soma and processes (Fig. 3*F*). These results corroborate our previously published findings that NHE6-null neurons display lower intra-endosomal (Ouyang et al., 2013); however, here, we extend our studies to demonstrate a more acidic pH in the lumen of lysosomes in the absence of NHE6.

### NHE6-null neurons have abnormal active CatD distribution across endosome and lysosome compartments

CatD is trafficked to lysosomes in various enzymatically inactive forms from the Golgi complex to endosomes until reaching the highly acidic lysosomal lumen where it is converted to its active form in a pH-dependent fashion (Zaidi et al., 2008). CatD is generally trafficked by the M6PR pathway (von Figura and Hasilik, 1986), although M6PR-independent routes have been reported (Gopalakrishnan et al., 2004; Canuel et al., 2008). Since NHE6-null primary neurons exhibit deficiencies in the enzymatically active CatD, we investigated whether this was because of impaired trafficking and/or distribution of CatD in the endosome and lysosome compartment. Given our prior data demonstrating overacidification of endosomal pH in NHE6-null neurons, we hypothesized that CatD may undergo precocious pH-dependent activation.

**Figure 4.**
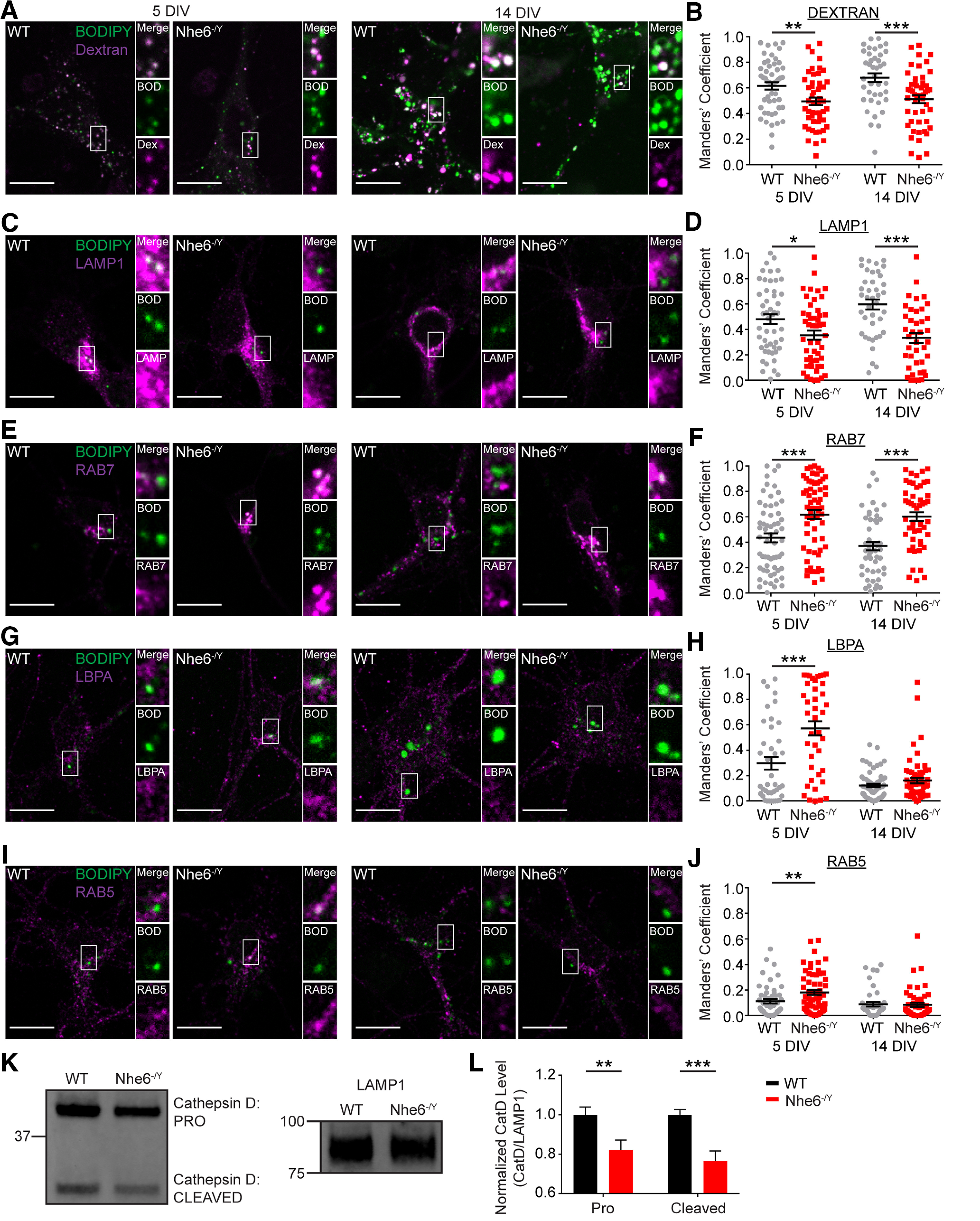
Loss of NHE6 alters CatD activation and distribution across the endosome and lysosome compartment. Confocal microscopy single-plane images of BODIPY-pepstatin A colocalization with different endosomes and the lysosome in male WT and NHE6-null mouse primary hippocampal neurons at DIV 5 and 14. Colocalization of active CatD within distinct endosome and lysosome compartments was tested using BODIPY-pepstatin A colocalization with the following markers: (***A***) dextran (lysosome), (***C***) LAMP1 (lysosome and late endosome), (***E***) RAB7 (late endosome), (***G***) LBPA (late endosome), and (***I***) RAB5 (early endosome). BODIPY-pepstatin A colocalization with these markers was quantified using the Manders' coefficient (i.e., degree BODIPY-pepstatin A signal overlaps with marker signal or M1). ***B***, Quantification of BODIPY-pepstatin A colocalization with dextran at 5 DIV (WT *n* = 50 cells, *Nhe6^-/Y^ n* = 50 cells, 5 mice per genotype, 3 litters, *p* = 0.004, Cohen's *d* = 0.58) and 14 DIV (WT *n* = 40 cells from 4 mice, *Nhe6^-/Y^ n* = 50 cells from 5 mice, 3 litters, *p* = 0.0004, Cohen's *d* = 0.78). ***D***, Quantification of BODIPY-pepstatin A colocalization with LAMP1 at 5 DIV (WT *n* = 50 cells, *Nhe6^-/Y^ n* = 50 cells, 5 mice per genotype, 4 litters, *p* = 0.018, Cohen's *d* = 0.48) and 14 DIV (WT *n* = 40 cells, *Nhe6^-/Y^ n* = 40 cells, 4 mice per genotype, 3 litters, *p* < 0.0001, Cohen's *d* = 1.06). ***F***, Quantification of BODIPY-pepstatin A colocalization with RAB7 at 5 DIV (WT *n* = 60 cells, *Nhe6^-/Y^ n* = 60 cells, 6 mice per genotype, 4 litters, *p* = 0.0004, Cohen's *d* = 0.66) and 14 DIV (WT *n* = 50 cells, *Nhe6^-/Y^ n* = 50 cells, 5 mice per genotype, 3 litters, *p* < 0.0001, Cohen's *d* = 0.95). ***H***, Quantification of BODIPY-pepstatin A colocalization with LBPA at 5 DIV (WT *n* = 40 cells, *Nhe6^-/Y^ n* = 40 cells, 4 mice per genotype, 3 litters, *p* < 0.0003, Cohen's *d* = 0.82) and 14 DIV (WT *n* = 70 cells, *Nhe6^-/Y^ n* = 70 cells, 7 mice per genotype, 5 litters). ***J***, Quantification of BODIPY-pepstatin A colocalization with RAB5 at 5 DIV (WT *n* = 50 cells from 5 mice, *Nhe6^-/Y^ n* = 60 cells from 6 mice, 4 litters, *p* = 0.009, Cohen's *d* = 0.49) and 14 DIV (WT *n* = 40 cells from 4 mice, *Nhe6^-/Y^ n* = 50 cells from 5 mice, 3 litters). ***K***, ***L***, CatD western blot (***K***) and quantification (***L***) in LEFs from acutely dissected 4-month-old WT and *Nhe6^-/Y^* male littermate hippocampus and neocortex combined (WT *n* = 10, *Nhe6^-/Y^ n* = 8, 7 litters, pro CatD *p* = 0.007, Hedges' *g* = 1.26, cleaved CatD *p* = 0.0007, Hedges' *g* = 1.99). CatD was normalized to LAMP1. Scale bars, 10 µm. BOD, BODIPY-pepstatin A; DEX, Dextran. Data are mean ± SEM. Unpaired two-tailed Student's *t* test (***B***,***D***,***F***, 14 DIV; ***L***, cleaved CatD) or Mann–Whitney test (***F***, 5 DIV; ***H***,***J***,***L***, pro-CatD).

**Figure 5.**
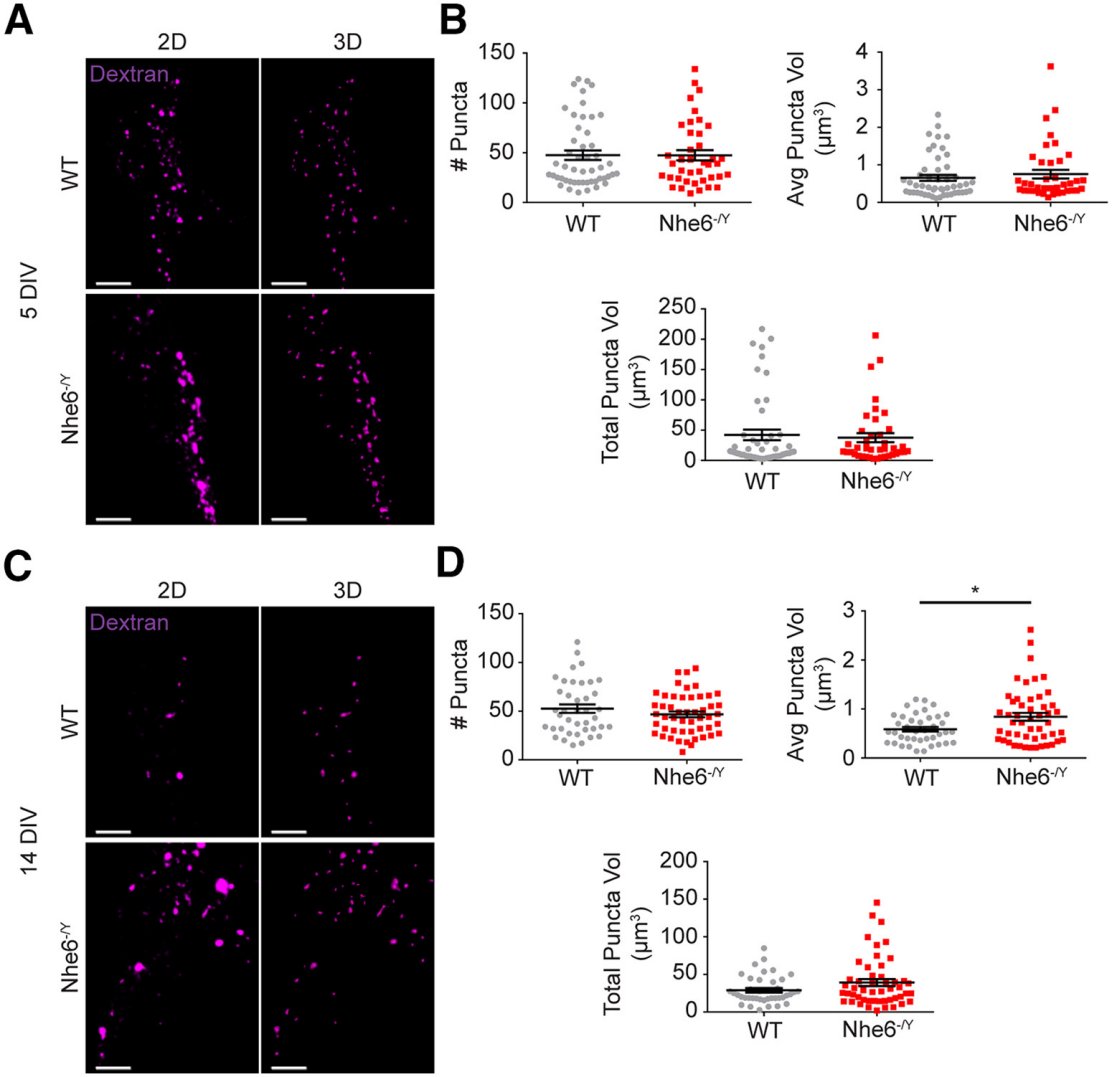
Larger lysosome-associated dextran puncta in mature NHE6-null neurons. ***A***, Confocal microscopy images of WT and *Nhe6^-/Y^* male primary hippocampal neurons following dextran treatment at DIV 5. Images are denoted as 2D (i.e., fluorescent microscopy images) and 3D (i.e., 3D reconstruction of dextran puncta). ***B***, Quantification of 3D-reconstructed dextran puncta at DIV 5 (WT *n* = 50 cells from 5 mice, *Nhe6^-/Y^ n* = 40 cells from 4 mice, 3 litters). Graphs depict the following: number of dextran puncta per cell, average dextran puncta volume per cell, and total summed dextran puncta volume per cell. ***C***, Confocal microscopy images of WT and *Nhe6^-/Y^* male primary hippocampal neurons following dextran treatment at DIV 14. ***D***, Quantification of 3D-reconstructed dextran puncta at DIV 14 (WT *n* = 40 cells from 4 mice, *Nhe6^-/Y^ n* = 50 cells from 5 mice, 3 litters). Graphs depict the following: number of dextran puncta per cell, average dextran puncta volume per cell (*p* = 0.04, Glass's Δ = 0.87), and total summed dextran puncta volume per cell (Welch's correction). Scale bars, 5 µm. Data are mean ± SEM. Unpaired two-tailed Student's *t* test (***D***, number of dextran puncta and average dextran puncta volume-transformed) or Mann–Whitney test (***B***,***D***, total dextran puncta volume).

**Figure 6.**
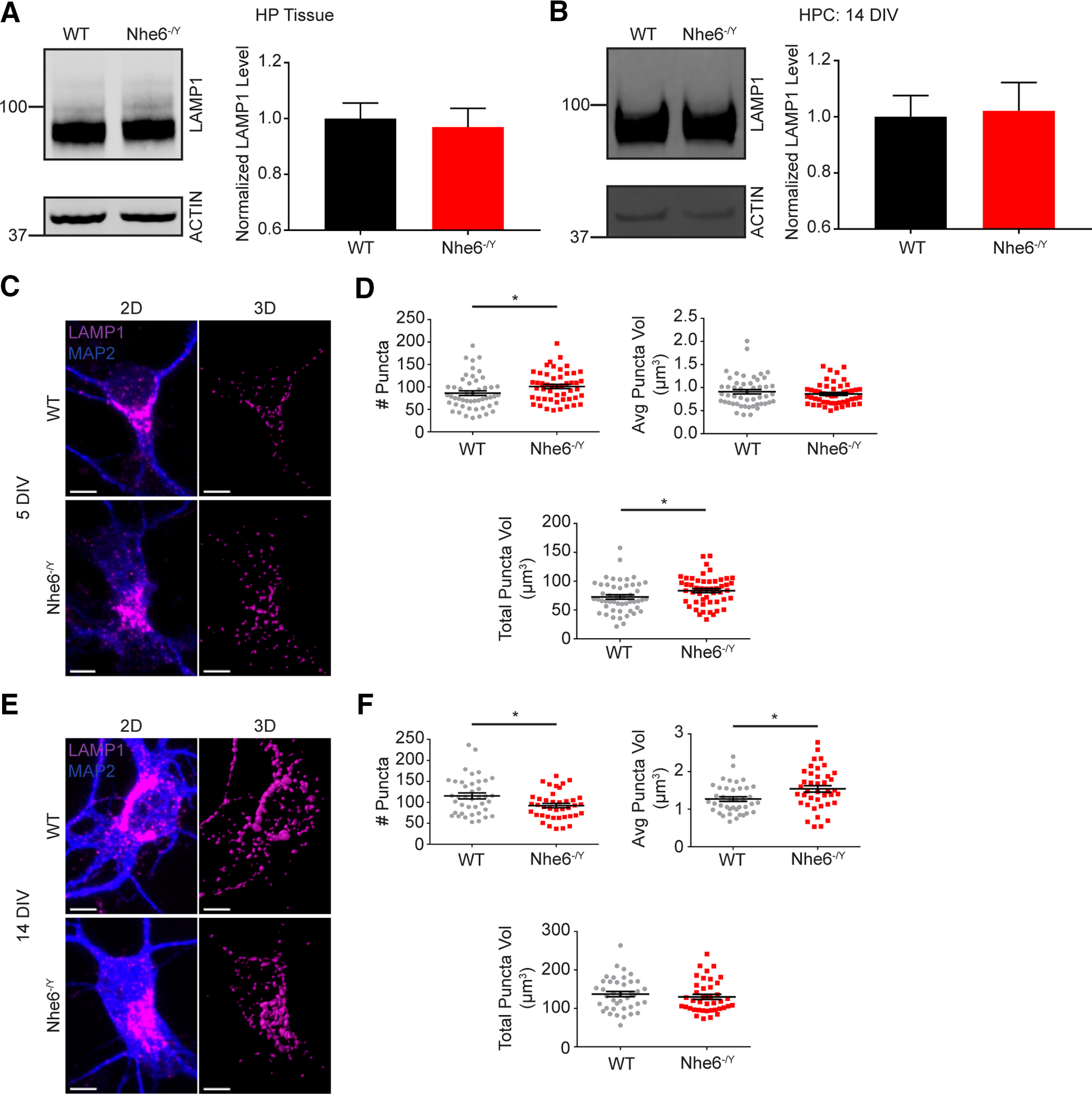
LAMP1 dysfunction in NHE6-null neurons. ***A***, LAMP1 western blot and quantification in WT and *Nhe6^-/Y^* male littermate mice, acutely dissected hippocampal tissue at 8 weeks old (WT *n* = 9 animals, *Nhe6^-/Y^ n* = 5 animals, 5 litters). ***B***, LAMP1 western blot and quantification in WT and *Nhe6^-/Y^* male primary hippocampal neurons at 14 DIV (WT *n* = 5 animals, *Nhe6^-/Y^ n* = 5 animals, 5 litters). ***C***, Confocal microscopy images of WT and *Nhe6^-/Y^* male primary hippocampal neurons at 5 DIV labeled with LAMP1 antibody. Images are denoted as 2D (i.e., fluorescent microscopy images) and 3D (i.e., 3D reconstruction of LAMP1 puncta). ***D***, Quantification of 3D-reconstructed LAMP1 puncta at DIV 5 (WT *n* = 50 cells, *Nhe6^-/Y^ n* = 50 cells, 5 mice per genotype, 3 litters). Graphs depict the following: number of LAMP1 puncta per cell (*p* = 0.02, Cohen's *d* = 0.42), average LAMP1 puncta volume per cell, and total summed LAMP1 puncta volume per cell (*p* = 0.03, Cohen's *d* = 0.42). ***E***, Confocal microscopy images of WT and *Nhe6^-/Y^* male primary hippocampal neurons at 14 DIV labeled with LAMP1 antibody. ***F***, Quantification of 3D-reconstructed LAMP1 puncta at DIV 14 (WT *n* = 40 cells, *Nhe6^-/Y^ n* = 40 cells, 4 mice per genotype, 3 litters). Graphs depict the following: number of LAMP1 puncta per cell (*p* = 0.01, Cohen's *d* = 0.58), average LAMP1 puncta volume per cell (*p* = 0.01, Glass's Δ = 0.69), and total summed LAMP1 puncta volume per cell. Scale bars, 5 µm. Data are mean ± SEM. Unpaired two-tailed Student's *t* test (***A***,***B***,***F***, number of LAMP1 puncta and total LAMP1 puncta volume) with Welch's correction (***F***, average LAMP1 puncta volume) or Mann–Whitney test (***D***).

We measured the subcellular distribution of active CatD using BODIPY FL pepstatin A with different endosome and lysosome markers. To reliably label lysosomes, primary hippocampal neurons were treated with fluorescent dextran and chased overnight. NHE6-null neurons exhibited significantly less active CatD-dextran colocalization compared with WT littermate controls at DIV 5 and 14 (Fig. 4*A*,*B*). This result reflects less active CatD in lysosome compartments in NHE6-null neurons, with increasing effect size with days in culture. We do not believe this apparent reduction in colocalization is because of alterations in the size or distribution of lysosome compartment alone. Using 3D volumetric reconstruction of dextran puncta, we observed no differences at 5 DIV (Fig. 5), a time at which we see decreases in colocalization of the mutant. We also found reduced active CatD colocalization using another lysosome-associated marker LAMP1 whereby NHE6-null neurons showed decreased active CatD-LAMP1 colocalization at DIV 5 and 14, again with increasing effect size with time in culture (Fig. 4*C*,*D*). However, we are aware that LAMP1 labels both degradative lysosomes as well as nondegradative organelles of endosomal and autophagic origin (Gowrishankar et al., 2015; Cheng et al., 2018; Kulkarni and Maday, 2018; Yap et al., 2018). Again, we do not believe that this pattern of active CatD is caused solely by alterations in lysosome size or distribution. There were no differences in LAMP1 protein levels in the mutant (Fig. 6*A*,*B*). 3D volumetric reconstruction revealed that NHE6-null neurons had significantly more LAMP1 puncta and total LAMP1 volume at 5 DIV (Fig. 6*C*,*D*). However, we observed significantly fewer LAMP1 puncta and greater average LAMP1 puncta volume at 14 DIV (Fig. 6*E*,*F*).

**Figure 7.**
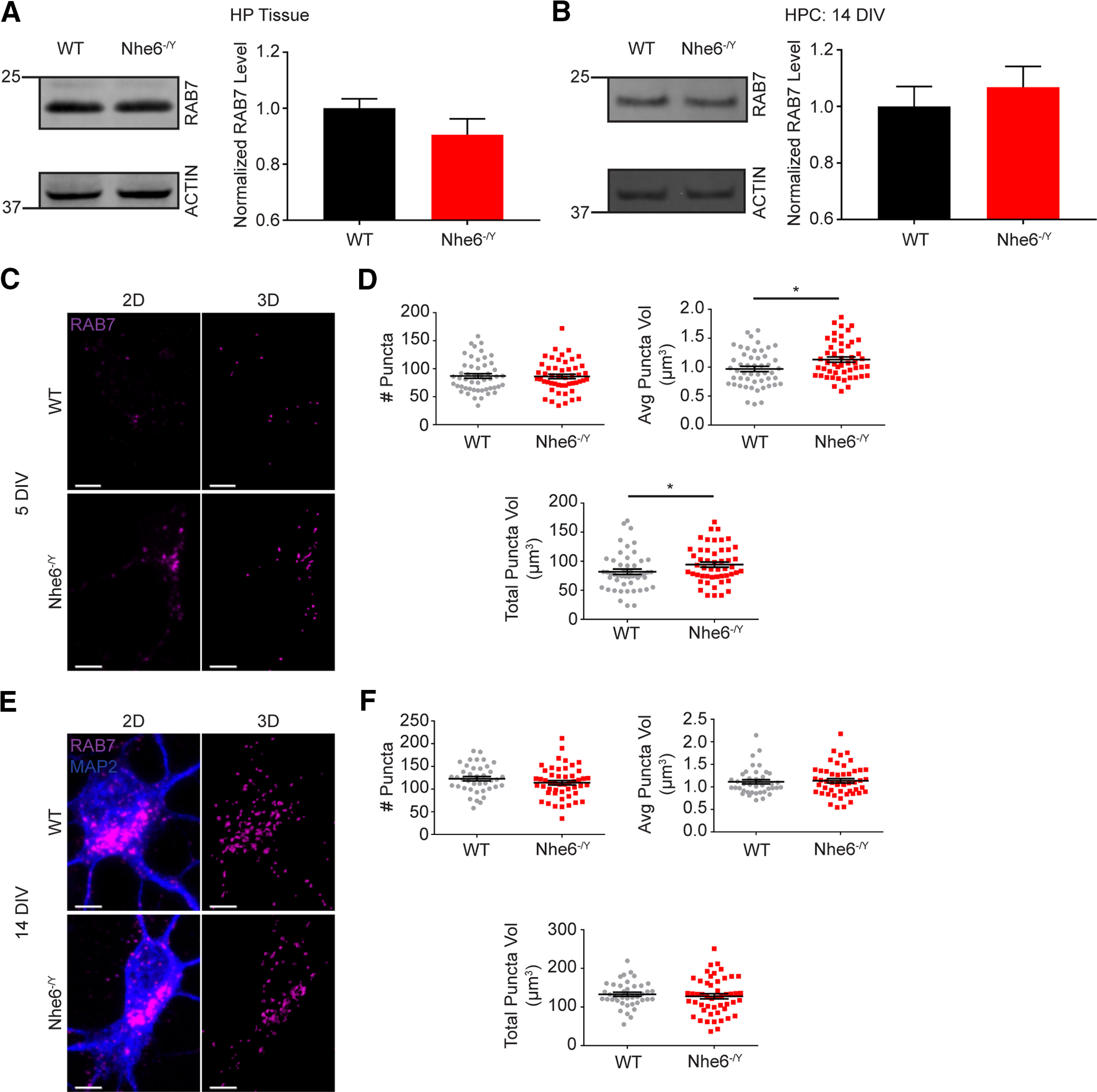
Greater RAB7 puncta volume in NHE6-null neurons. ***A***, RAB7 western blot and quantification in WT and *Nhe6^-/Y^* male littermate mice, acutely dissected hippocampal tissue at 8 weeks old (WT *n* = 11 animals, *Nhe6^-/Y^ n* = 6 animals, 6 litters). ***B***, RAB7 western blot and quantification in WT and *Nhe6^-/Y^* male primary hippocampal neurons at 14 DIV (WT *n* = 5 animals, *Nhe6^-/Y^ n* = 5 animals, 5 litters). ***C***, Confocal microscopy images of WT and *Nhe6^-/Y^* male primary hippocampal neurons at 5 DIV labeled with RAB7 antibody. Images are denoted as 2D (i.e., fluorescent microscopy images) and 3D (i.e., 3D reconstruction of RAB7 puncta). ***D***, Quantification of 3D-reconstructed RAB7 puncta at DIV 5 (WT *n* = 50 cells, *Nhe6^-/Y^ n* = 50 cells, 5 mice per genotype, 3 litters). Graphs depict the following: number of RAB7 puncta per cell, average RAB7 puncta volume per cell (*p* = 0.01, Cohen's *d* = 0.51), and total summed RAB7 puncta volume per cell (*p* = 0.04, Cohen's *d* = 0.38). ***E***, Confocal microscopy images of WT and *Nhe6^-/Y^* male primary hippocampal neurons at 14 DIV labeled with RAB7 antibody. ***F***, Quantification of 3D-reconstructed RAB7 puncta at DIV 14 (WT *n* = 40 cells from 4 mice, *Nhe6^-/Y^ n* = 50 cells from 5 mice, 3 litters). Graphs depict the following: number of RAB7 puncta per cell, average RAB7 puncta volume per cell, and total summed RAB7 puncta volume per cell. Scale bars, 5 µm. Data are mean ± SEM. Unpaired two-tailed Student's *t* test (***A***,***B***,***D***, number of RAB7 puncta and average RAB7 puncta volume; ***F***, RAB7 puncta) with Welch's correction (***F***, total RAB7 puncta volume) or Mann–Whitney test (***D***, total RAB7 puncta volume; ***F***, average RAB7 puncta volume).

**Figure 8.**
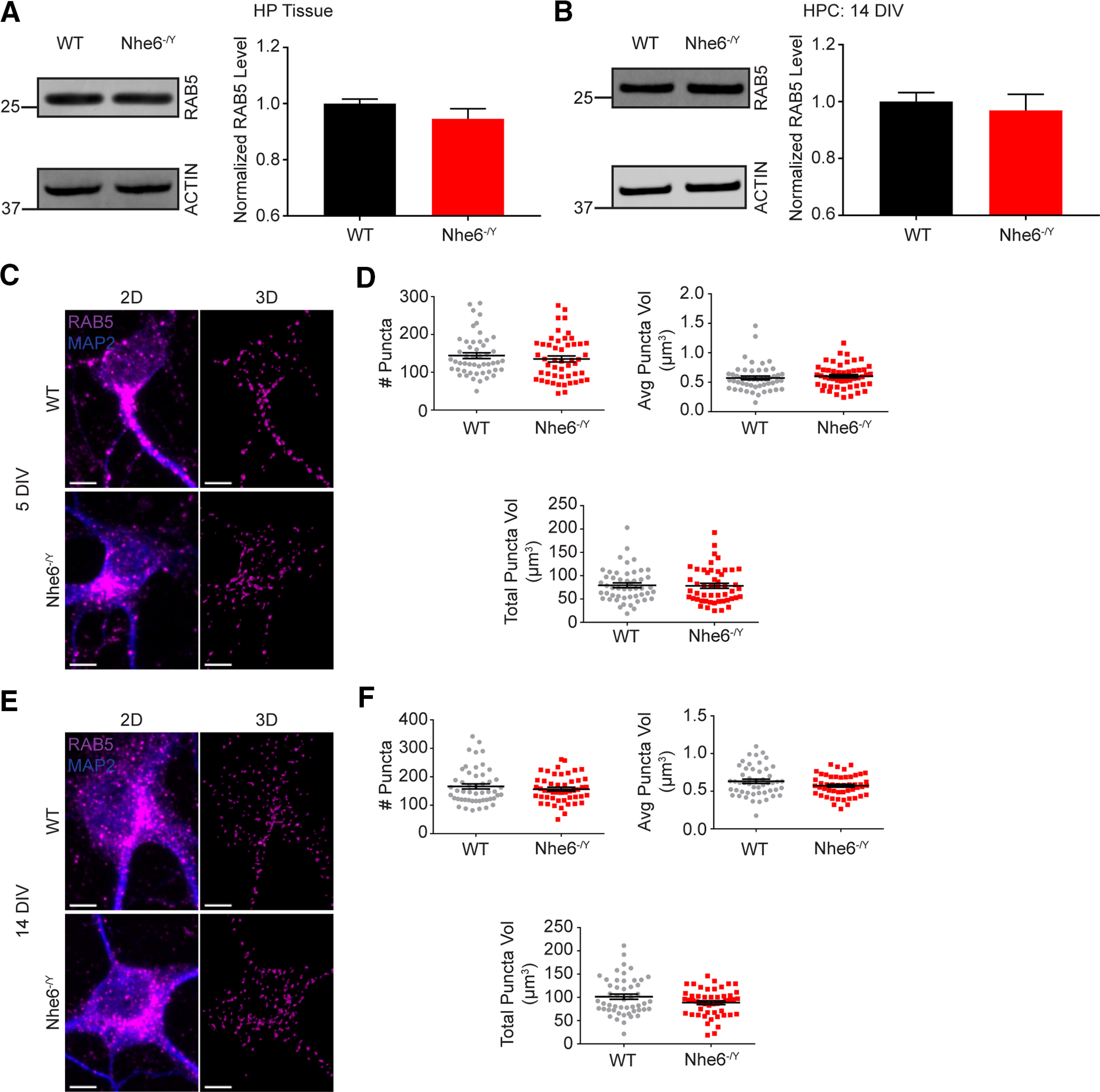
RAB5 features unaffected by loss of NHE6. ***A***, RAB5 western blot and quantification in WT and *Nhe6^-/Y^* male littermate mice, acutely dissected hippocampal tissue at 8 weeks old (WT *n* = 9 animals, *Nhe6^-/Y^ n* = 6 animals, 6 litters). ***B***, RAB5 western blot and quantification in WT and *Nhe6^-/Y^* male primary hippocampal neurons at 14 DIV (WT *n* = 5 animals, *Nhe6^-/Y^ n* = 5 animals, 5 litters). ***C***, Confocal microscopy images of WT and *Nhe6^-/Y^* male primary hippocampal neurons at 5 DIV labeled with RAB5 antibody. Images are denoted as 2D (i.e., fluorescent microscopy images) and 3D (i.e., 3D reconstruction of RAB5 puncta). ***D***, Quantification of 3D-reconstructed RAB5 puncta at DIV 5 (WT *n* = 50 cells, *Nhe6^-/Y^ n* = 50 cells, 5 mice per genotype, 4 litters). Graphs depict the following: number of RAB5 puncta per cell, average RAB5 puncta volume per cell, and total summed RAB5 puncta volume per cell. ***E***, Confocal microscopy images of WT and *Nhe6^-/Y^* male primary hippocampal neurons at 14 DIV labeled with RAB5 antibody. ***F***, Quantification of 3D-reconstructed RAB5 puncta at DIV 14 (WT *n* = 50 cells, *Nhe6^-/Y^ n* = 50 cells, 5 mice per genotype, 3 litters). Graphs depict the following: number of RAB5 puncta per cell, average RAB5 puncta volume per cell, and total summed RAB5 puncta volume per cell. Scale bars, 5 µm. Data are mean ± SEM. Unpaired two-tailed Student's *t* test (***A***,***B***) with Welch's correction (***F***, average RAB5 puncta volume and total RAB5 puncta volume) or Mann–Whitney test (***D***,***F***, number of RAB5 puncta).

By contrast to the findings of reduced active CatD in mutant lysosomes, NHE6-null neurons displayed significantly greater colocalization of active CatD within the early and late endosome compartment. The late endolysosome compartment was initially studied using the marker RAB7 at DIV 5 and 14, wherein there was more active CatD (Fig. 4*E*,*F*). There were no differences in RAB7 protein levels, yet NHE6-null neurons had larger RAB7 puncta volume and more total RAB7 volume at DIV 5 (Fig. 7). We examined active CatD colocalization with LBPA, an atypical phospholipid found on the internal membrane of late endosomes (Kobayashi et al., 1998). Active CatD colocalization with LBPA was enhanced at DIV 5 relative to control, and although not statistically significant, showed greater colocalization at DIV 14 (Fig. 4*G*,*H*). Also, NHE6-null neurons exhibited significantly greater active CatD colocalization with the early endosome marker RAB5, at DIV 5 but not DIV 14 (Fig. 4*I*,*J*). There were no differences in RAB5 protein levels or puncta features in mutant neurons (Fig. 8). These endosome results are consistent with overacidification of the endosome lumen (Fig. 3*E*,*F*) and precocious activation of CatD; however, perhaps with a compensatory response in the early endosome by later stages of culturing.

Our data support a reduced level and/or activity of CatD in the lysosome, suggesting that loss of NHE6 impairs CatD trafficking to lysosomes. To further corroborate our lysosome findings, we measured CatD protein levels directly within the lysosome by cellular fractionation using lysosome-enriched fractions (LEFs) collected *ex vivo* from 4-month-old CS mouse hippocampal and neocortical tissue combined. LEFs from NHE6-null mice had significantly decreased pro- and cleaved-CatD levels compared with male littermate controls (Fig. 4*K*,*L*). These results show strong effective sizes (Hedges' *g* = 1.26 for pro-CatD and Hedges' *g* = 1.99 for cleaved). Collectively, these findings indicate that loss of NHE6 causes two important effects on CatD: (1) premature CatD activity in earlier stages in the endocytic pathway and (2) impaired trafficking of CatD to lysosomes.

### Loss of NHE6 alters M6PR distribution

**Figure 9.**
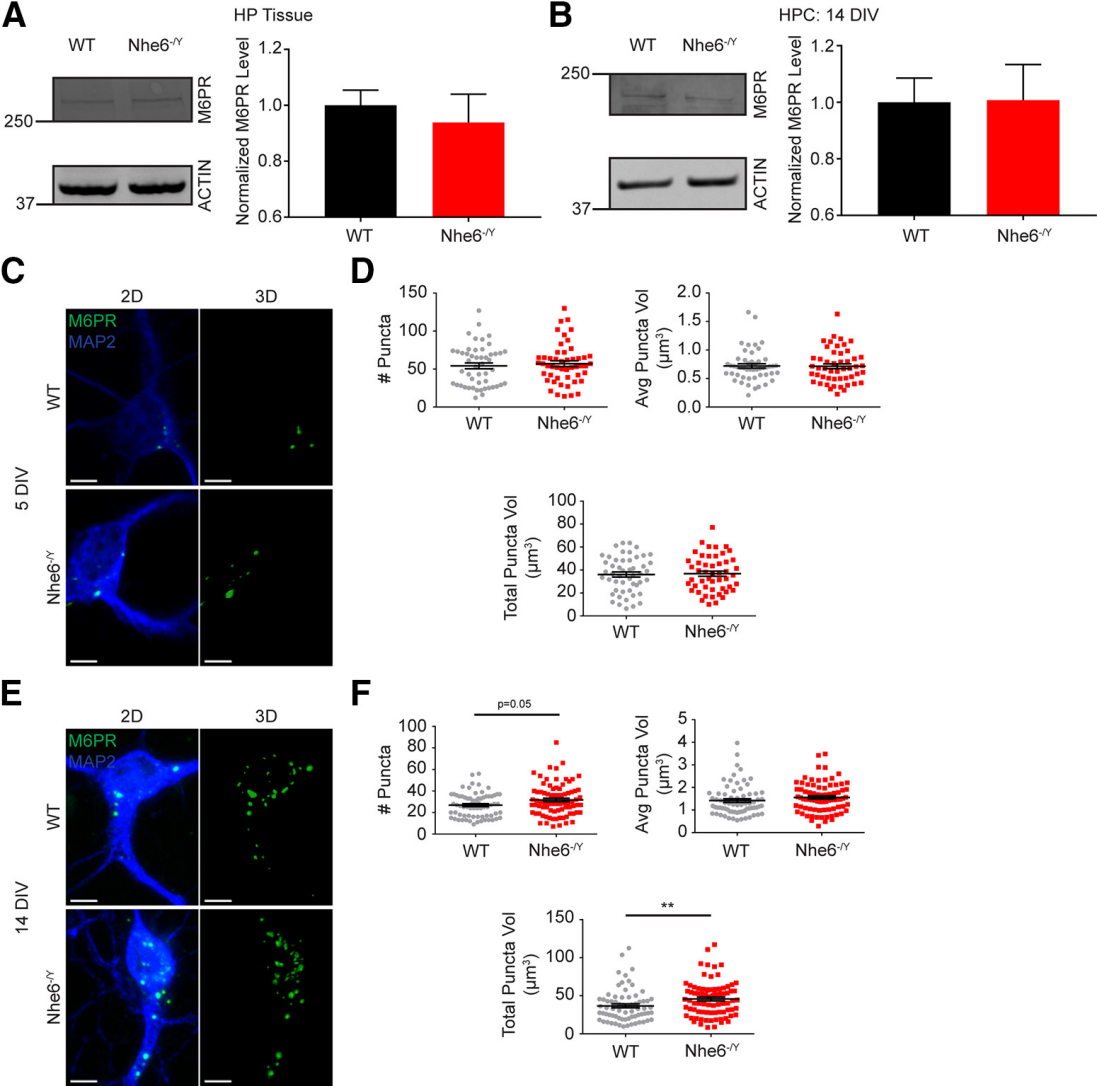
Greater M6PR puncta volume in mature NHE6-null neurons. ***A***, M6PR western blot and quantification in WT and *Nhe6^-/Y^* male littermate mice, acutely dissected hippocampal tissue at 8 weeks old (WT *n* = 11 animals, *Nhe6^-/Y^ n* = 6 animals, 6 litters). ***B***, M6PR western blot and quantification in WT and *Nhe6^-/Y^* male primary hippocampal neurons at 14 DIV (WT *n* = 4 animals, *Nhe6^-/Y^ n* = 4 animals, 4 litters). ***C***, Confocal microscopy images of WT and *Nhe6^-/Y^* male primary hippocampal neurons at 5 DIV labeled with M6PR antibody. Images are denoted as 2D (i.e., fluorescent microscopy images) and 3D (i.e., 3D reconstruction of M6PR puncta). ***D***, Quantification of 3D-reconstructed M6PR puncta at DIV 5 (WT *n* = 50 cells, *Nhe6^-/Y^ n* = 50 cells, 5 mice per genotype, 3 litters). Graphs depict the following: number of M6PR puncta per cell, average M6PR puncta volume per cell, and total summed M6PR puncta volume per cell. ***E***, Confocal microscopy images of WT and *Nhe6^-/Y^* male primary hippocampal neurons at 14 DIV labeled with M6PR antibody. ***F***, Quantification of 3D-reconstructed M6PR puncta at DIV 14 (WT *n* = 70 cells from 7 mice, *Nhe6^-/Y^ n* = 80 cells from 8 mice, 6 litters). Graphs depict the following: number of M6PR puncta per cell (*p* = 0.05, Glass's Δ = 0.44), average M6PR puncta volume per cell, and total summed M6PR puncta volume per cell (*p* = 0.004, Cohen's *d* = 0.41). Scale bars, 5 µm. Data are mean ± SEM. Unpaired two-tailed Student's *t* test (***A***,***B***,***D***, number of M6PR puncta and total M6PR puncta volume) or Mann–Whitney test (***D***, average M6PR puncta volume, ***F***).

Given our findings that M6PR-dependent lysosomal enzymes (i.e., CatD and β-NAG) are notably affected in NHE6-null neurons, we investigated whether this was due, in part, to impaired trafficking of M6PRs. M6PR shuttles between the TGN and the endocytic pathway (Brown et al., 1986; Hirst et al., 1998; Ghosh et al., 2003; Lin et al., 2004; Kucera et al., 2016). Before reaching lysosomes, M6PRs are retrogradely transported back to the TGN via the retromer complex (e.g., VPS26, VPS29, and VPS35) (Arighi et al., 2004; Seaman, 2004; Cui et al., 2019). Notably, we did not observe differences in M6PR protein levels by western blot between NHE6 null and control neurons (Fig. 9*A*,*B*); however, NHE6-null neurons had significantly greater M6PR puncta numbers and total volume per cell at 14 DIV (Fig. 9*E*,*F*).

**Figure 10.**
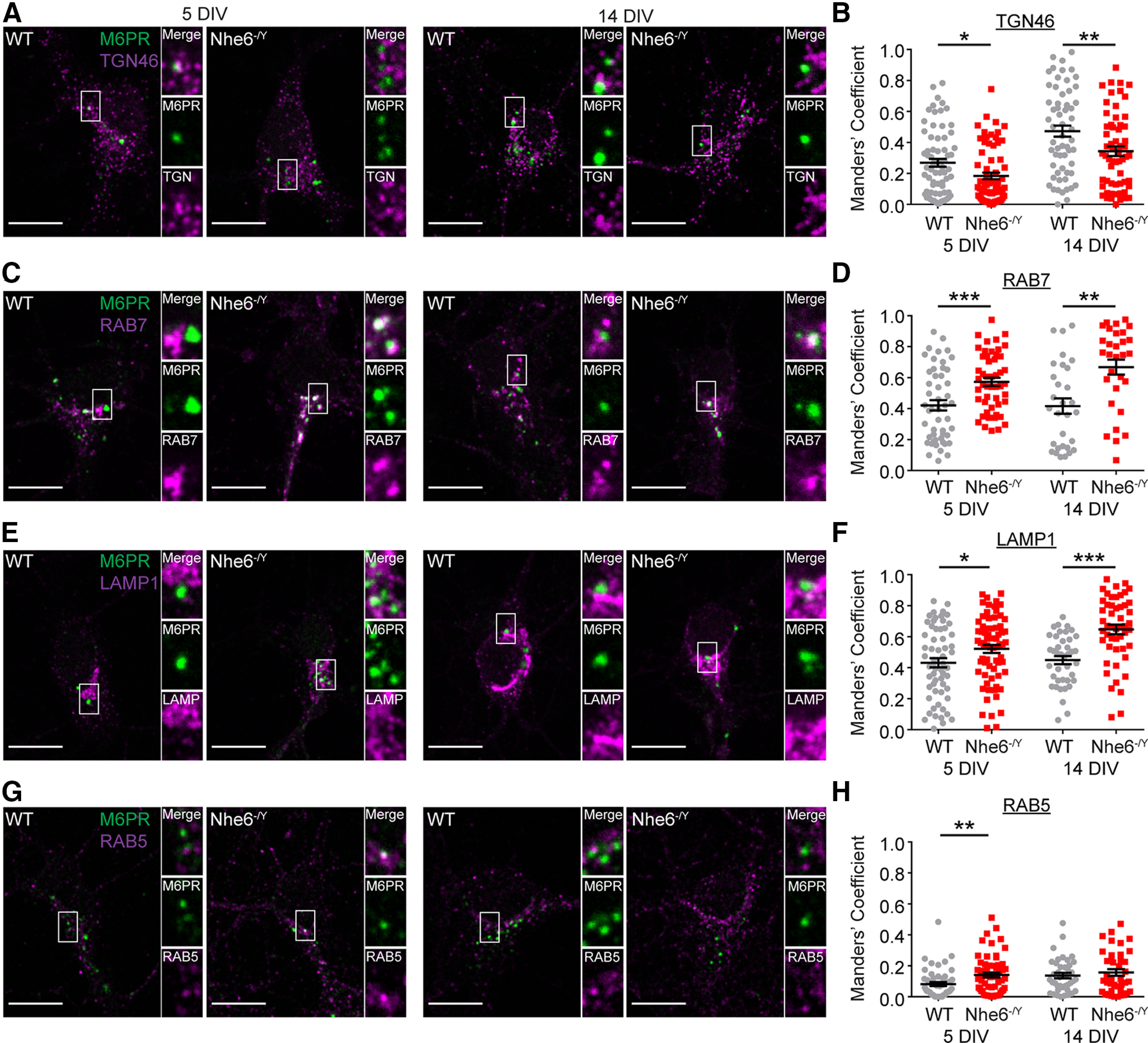
Altered M6PR distribution in NHE6-null neurons *in vitro*. Confocal microscopy single-plane images of M6PR colocalization with different markers of the endocytic pathway in male WT and NHE6-null mouse primary hippocampal neurons at DIV 5 and 14. Colocalization of M6PR was tested using the following markers: (***A***) TGN46 (TGN), (***C***) RAB7 (late endosome), (***E***) LAMP1 (late endosome), and (***G***) RAB5 (early endosome). M6PR colocalization with these markers was quantified using the Manders' coefficient (i.e., degree M6PR signal overlaps with marker signal or M1). ***B***, Quantification of M6PR colocalization with TGN46 at 5 DIV (WT *n* = 70 cells, *Nhe6^-/Y^ n* = 70 cells, 7 mice per genotype, 4 litters, *p* = 0.01, Cohen's *d* = 0.43) and 14 DIV (WT *n* = 60 cells from 6 mice, *Nhe6^-/Y^ n* = 60 cells from 6 mice, 4 litters, *p* = 0.009, Cohen's *d* = 0.50). ***D***, Quantification of M6PR with RAB7 at 5 DIV (WT *n* = 50 cells, *Nhe6^-/Y^ n* = 50 cells, 5 mice per genotype, 3 litters, *p* = 0.0008, Cohen's *d* = 0.71) and 14 DIV (WT *n* = 40 cells, *Nhe6^-/Y^ n* = 40 cells, 4 mice per genotype, 3 litters, *p* = 0.003, Cohen's *d* = 0.72). ***F***, Quantification of M6PR with LAMP1 at 5 DIV (WT *n* = 60 cells from 6 mice, *Nhe6^-/Y^ n* = 70 cells from 7 mice, 4 litters, *p* = 0.03, Cohen's *d* = 0.40) and 14 DIV (WT *n* = 40 cells from 4 mice, *Nhe6^-/Y^ n* = 50 cells from 5 mice, 3 litters, *p* < 0.0001, Cohen's *d* = 1.03). ***H***, Quantification of M6PR colocalization with RAB5 at 5 DIV (WT *n* = 50 cells from 5 mice, *Nhe6^-/Y^ n* = 60 cells from 6 mice, 4 litters, *p* = 0.009, Glass's Δ = 0.64) and 14 DIV (WT *n* = 40 cells, *Nhe6^-/Y^ n* = 40 cells, 4 mice per genotype, 2 litters). Scale bars, 10 µm. Data are mean ± SEM. Unpaired two-tailed Student's *t* test (***F***, 14 DIV) or Mann–Whitney test (***B***,***D***,***F***, 5 DIV, ***H***).

To investigate the steady-state distribution of endogenous cation-independent-M6PR (CI-M6PR), we measured M6PR colocalization with markers for the TGN and endolysosome compartments. NHE6-null primary hippocampal neurons exhibited significantly decreased M6PR colocalization with the TGN marker TGN46 at DIV 5 and 14 (Fig. 10*A*,*B*). On the other hand, NHE6-null neurons showed significantly greater M6PR-RAB7 colocalization at DIV 5 and 14, reflecting enhanced distribution of M6PRs in the late endosomes in NHE6-null neurons (Fig. 10*C*,*D*). Furthermore, NHE6-null neurons demonstrated significantly greater M6PR-LAMP1 colocalization at DIV 5 and 14 (Fig. 10*E*,*F*). NHE6-null neurons also had significantly greater M6PR-RAB5 colocalization at DIV 5, but not DIV 14 (Fig. 10*G*,*H*). Taken together, these findings are consistent with altered M6PR trafficking in NHE6-null neurons, perhaps reflecting defects in retrograde trafficking of M6PR back to Golgi.

### NHE6-null neurons have diminished endosome-lysosome fusion

**Figure 11.**
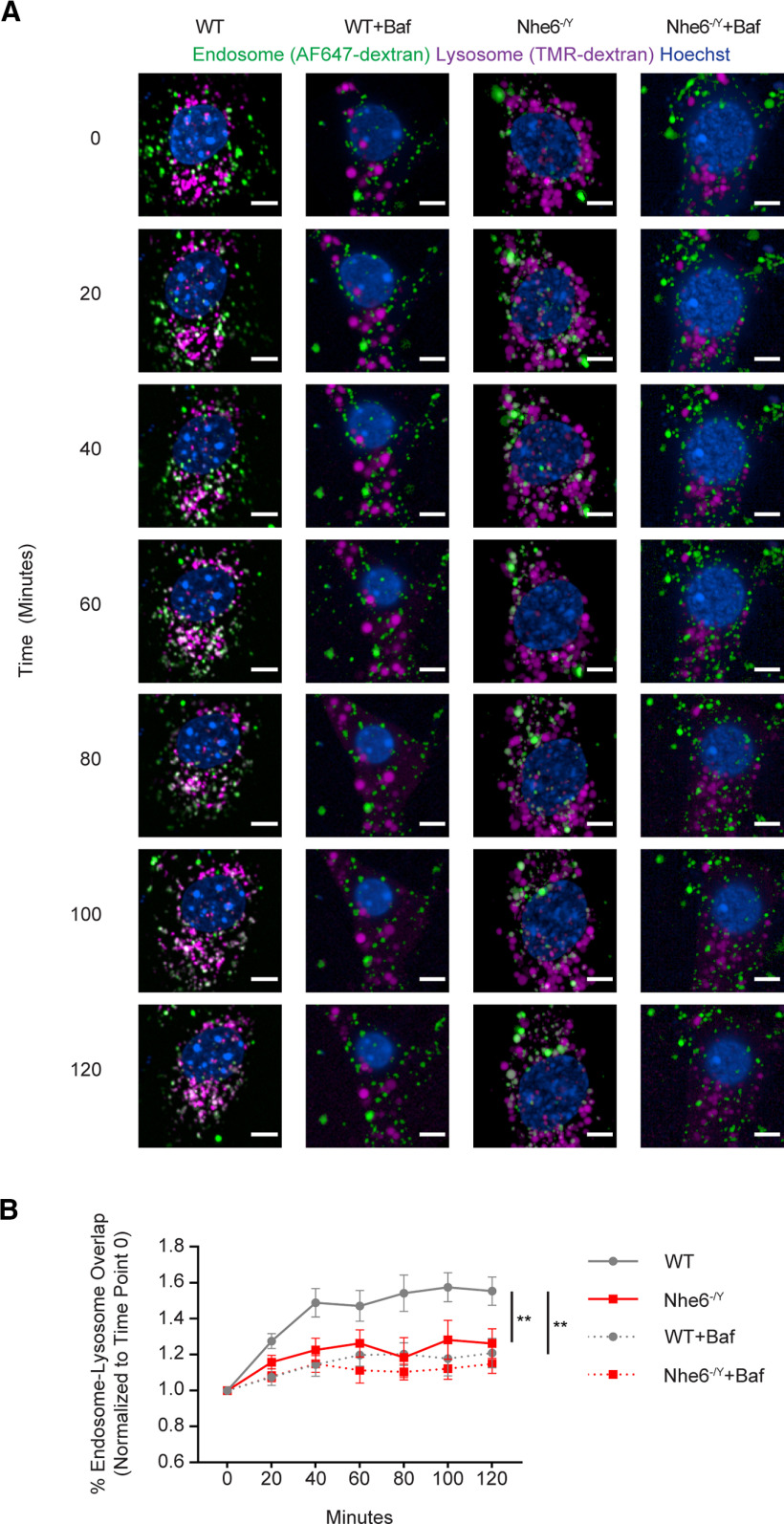
Delayed endosome-lysosome fusion in NHE6-null neurons *in vitro*. ***A***, Live-cell confocal microscopy imaging of endosome-lysosome fusion in WT and *Nhe6^-/Y^* male mouse primary hippocampal neurons at 5 DIV with and without bafilomycin A treatment. The following time points were measured following incubation with AlexaFluor-647-dextran: 0, 20, 40, 60, 80, 100, and 120 min. ***B***, Quantification of endosome-lysosome fusion (WT *n* = 7 animals, *Nhe6^-/Y^ n* = 7 animals, 5 litters). Endosome-lysosome fusion % is expressed as % fold change to time point 0 for the same animal. There was a significant interaction effect for time × genotype (*F*_(6,72.0)_ = 3.432, *p* = 0.005). Scale bars, 5 µm. Data are mean ± SEM. Linear mixed model.

Loss of Nhx1 in *S. cerevisiae*, the NHE6 homolog in yeast, impairs MVB fusion with vacuoles (i.e., the lysosome equivalent in yeast) (Karim and Brett, 2018). Given these observations and our data on reduced CatD activity in lysosomes in neurons, we set out to measure endosome-lysosome fusion in primary hippocampal neurons. Primary neurons were incubated with TMR-dextran on DIV 4 to allow for trafficking to lysosomes. On DIV 5, cells were briefly incubated with AlexaFluor-647-dextran for 10 min for internalization via endocytosis. Time-lapse images collected every 20 min over the span of 2 h captured endosome-lysosome fusion events, as defined by endocytosed AlexaFluor-647-dextran puncta that colocalized with the lysosome marker TMR-dextran. NHE6-null neurons displayed significantly less endosome-lysosome fusion across all time points throughout a 2 h span compared to WT littermates (Fig. 11*A*,*B*). There was a statistically significant time × genotype interaction (*F*_(6,72.0)_ = 3.432, *p* = 0.005) (Fig. 11*B*). As a control, cells were treated with bafilomycin A, which disrupts the trafficking of late endosomes to lysosomes (van Weert et al., 1995). As expected, bafilomycin A treatment impaired endosome-lysosome fusion in WT and NHE6-null neurons. The kinetics of endosome-lysosome fusion between NHE6-null neurons without bafilomycin A and control neurons with bafilomycin A were similar. Our data therefore suggest that loss of NHE6 significantly impedes or delays endosome-lysosome fusion in neurons *in vitro*.

### Loss of NHE6 enhances MVB-PM fusion and exosome secretion

**Figure 12.**
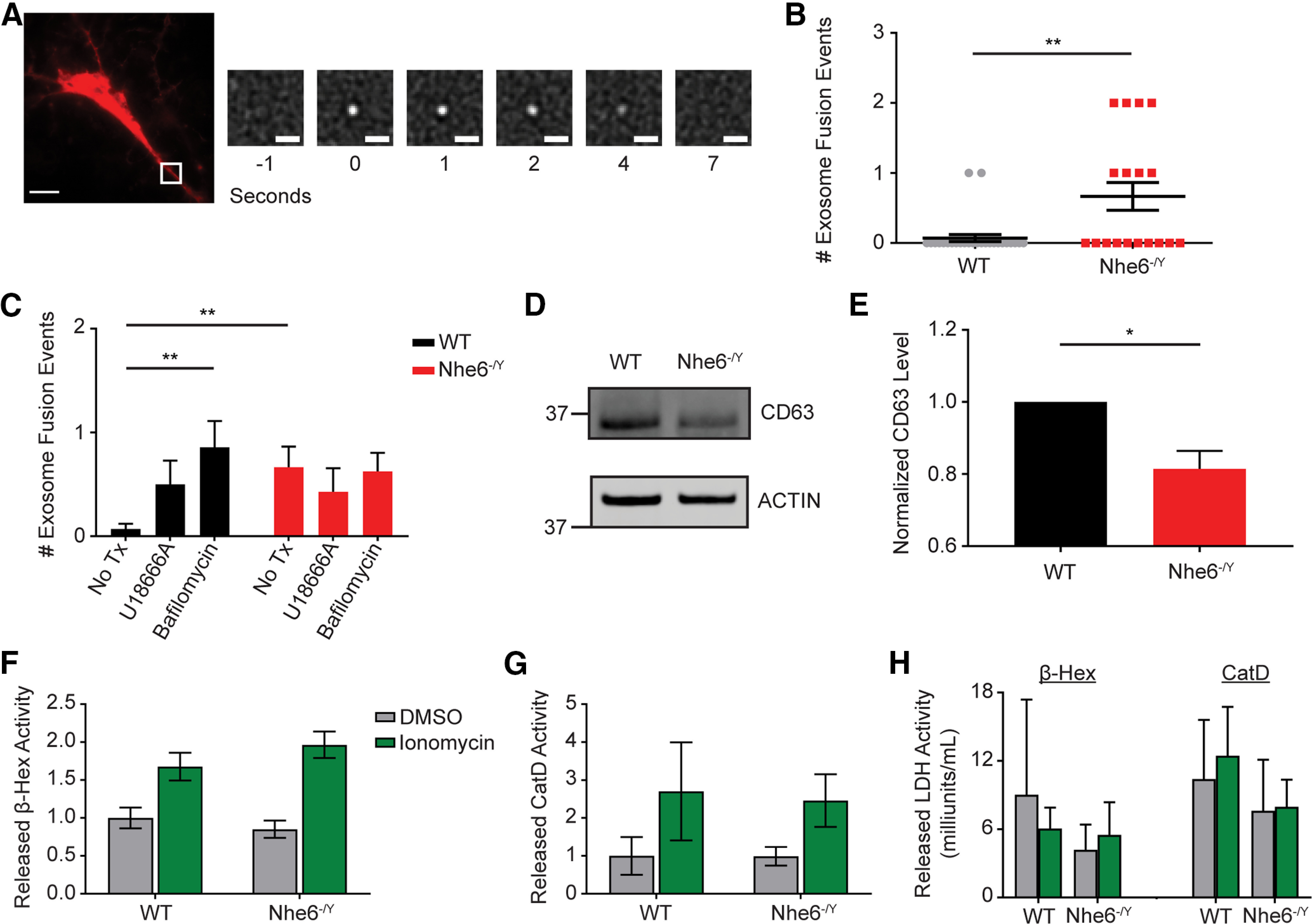
Loss of NHE6 increases MVB fusion with the PM and exosome secretion. ***A***, Representative TIRF image depicting MVB-PM fusion and exosome release as developed from Verweij et al. (2018). Widefield image of neuron cotransfected with mCherry and CD63-pHluorin expression constructs. White inset, Location of MVB-PM fusion and the zoomed in panels on the right. Each panel represents the progression of a CD63-pHluorin fusion event with the PM with the number of seconds indicated below the panel. Scale bars: large, 10 µm; small, 1 µm. ***B***, Quantification of full MVB-PM fusion/exosome release events per cell over 5 min in WT and *Nhe6^-/Y^* male littermate mouse primary hippocampal neurons at 14 DIV (WT *n* = 28 cells from 7 mice, *Nhe6^-/Y^ n* = 18 cells from 7 mice, 5 litters, *p* = 0.009, Glass's Δ = 2.27). ***C***, Quantification of full MVB-PM fusion/exosome release events per cell over 5 min in WT and *Nhe6^-/Y^* male mouse primary hippocampal neurons at 14 DIV under the following conditions: untreated (same as in ***B***), U18666A (positive control) (WT *n* = 14 cells from 5 mice, *Nhe6^-/Y^ n* = 14 cells from 5 mice, 3 litters), bafilomycin A1 (positive control) (WT *n* = 14 cells from 7 mice, *Nhe6^-/Y^ n* = 16 cells from 6 mice, 4 litters, Kruskal–Wallis test with Dunn's test: WT untreated compared with WT bafilomycin A1 *p* = 0.002, Glass's Δ = 3.04). ***D***, ***E***, CD63 western blot (***D***) and quantification (***E***) in WT and *Nhe6^-/Y^* male littermate mouse primary hippocampal neurons at 14 DIV (WT *n* = 5 cultures, *Nhe6^-/Y^ n* = 5 cultures, 5 litters, *p* = 0.02, Glass's Δ = 1.68). ***F***, Released β-Hex enzyme activity following short-term incubation in Tyrode's solution followed by treatment with either ionomycin or DMSO (WT *n* = 9, *Nhe6^-/Y^ n* = 9, 8 litters). ***G***, Released CatD enzyme activity following short-term incubation in Tyrode's solution followed by treatment with either ionomycin or DMSO (WT *n* = 6, *Nhe6^-/Y^ n* = 6, 5 litters). ***H***, Released LDH activity across all β-Hex (WT *n* = 5, *Nhe6^-/Y^ n* = 5, 5 litters) and CatD (WT *n* = 4, *Nhe6^-/Y^ n* = 4, 3 litters) experiments. Data are mean ± SEM. Unpaired two-tailed Student's *t* test (***C***, WT-*Nhe6^-/Y^*: bafilomycin A1) with Welch's correction (***E***), Mann–Whitney test (***B***,***C***, WT-*Nhe6^-/Y^*: U18666A), Kruskal–Wallis test with Dunn's test (***C***, differences between treatments by genotype), two-way ANOVA with Tukey's multiple comparisons test (***F***,***G***,***H***).

Given impaired endosome to lysosome trafficking, we investigated whether loss of NHE6 altered the trafficking of late endosomes/MVBs. We used a CD63-pHluorin construct to visualize MVB fusion with the PM in live neurons using TIRF microscopy (Verweij et al., 2018; Bebelman et al., 2020). This construct is also able to visualize exosomes, a class of extracellular vesicles originating from endosomes (Colombo et al., 2014). Interestingly, endosomal acidification has been identified as a key regulator of exosome release (Parolini et al., 2009; Bonsergent and Lavieu, 2019; Bonsergent et al., 2021). In this experiment, individual MVB-PM fusion/exosome secretion events were scored in primary hippocampal neurons at 14 DIV (Fig. 12*A*; Movie 1). To reliably identify neurons transfected with the CD63-pHluorin, we cotransfected an mCherry vector to label neurons. Control neurons demonstrated a remarkably low level of MVB-PM fusion events; however, notably, NHE6-null neurons displayed significantly greater MVB-PM fusion/exosome secretion events compared with WT male littermate controls (Fig. 12*B*). Statistical analysis using an ordinal logistic regression revealed that NHE6-null neurons were more likely to exhibit multiple MVB-PM fusion/exosome secretion events (i.e., ≥2 events) than control neurons (odds ratio = 11.4; 95% CI = 1.9, 66.6; *p* = 0.007). As a positive control, we treated primary hippocampal neurons with bafilomycin A1 (Villarroya-Beltri et al., 2016) and U186668 (Strauss et al., 2010), each of which increases small- to medium-sized EV secretion. Bafilomycin A1 treatment significantly increased MVB-PM fusion/exosome secretion in WT neurons compared with untreated neurons (H_(2)_ = 12.44, *p* = 0.002, Kruskal–Wallis test with Dunn's test for multiple comparisons) (Fig. 12*C*). In NHE6-null neurons, bafilomycin A1 treatment did not increase MVB-PM fusion/exosome secretion above untreated mutant neurons, possibly because of the high fusion events at baseline. We also measured CD63 protein levels in primary hippocampal neurons at 14 DIV. Interestingly, there was significantly less CD63 in NHE6-null neurons (Fig. 12*D*,*E*). Taken together, these findings suggest that NHE6-null neurons display enhanced MVB-PM fusion and CD63-associated exosome secretion. Furthermore, it is unlikely that this reflects an increase in CD63 protein levels as CD63 protein levels were reduced in NHE6-null neurons, perhaps in part because of excess release of CD63-positive exosomes.

Movie 1. MVB fusion with the PM and exosome release. 10.1523/JNEUROSCI.1244-20.2021.video.1

We investigated whether loss of NHE6 also led to greater lysosome fusion with the PM, a Ca^2+^-dependent process known as lysosomal exocytosis (Andrews, 2000; Blott and Griffiths, 2002). Enhancing lysosomal exocytosis has been shown to promote the extracellular release of pathogenic substrates in various lysosomal storage diseases (Medina et al., 2011). To measure lysosomal exocytosis *in vitro*, we measured the activity of extracellularly secreted lysosome enzymes in primary hippocampal neurons at DIV 14. As a positive control, cells were treated with ionomycin, a calcium ionophore that increases cytoplasmic Ca^2+^ concentration, which enhances lysosomal exocytosis (Rodriguez et al., 1997). Importantly, there were no differences in released activity of the lysosomal enzyme β-Hex at baseline (i.e., DMSO) as well as following ionomycin treatment in NHE6-null neurons relative to control neurons (Fig. 12*F*). Similar results were found when measuring released CatD enzyme activity (Fig. 12*G*). There were no differences in cell death, as measured by released LDH enzyme activity, across all treatments (Fig. 12*H*). We therefore conclude that loss of NHE6 causes specific changes in MVB-PM fusion/CD63-positive exosome release that does not extend to lysosomal exocytosis.

## Discussion

**Figure 13.**
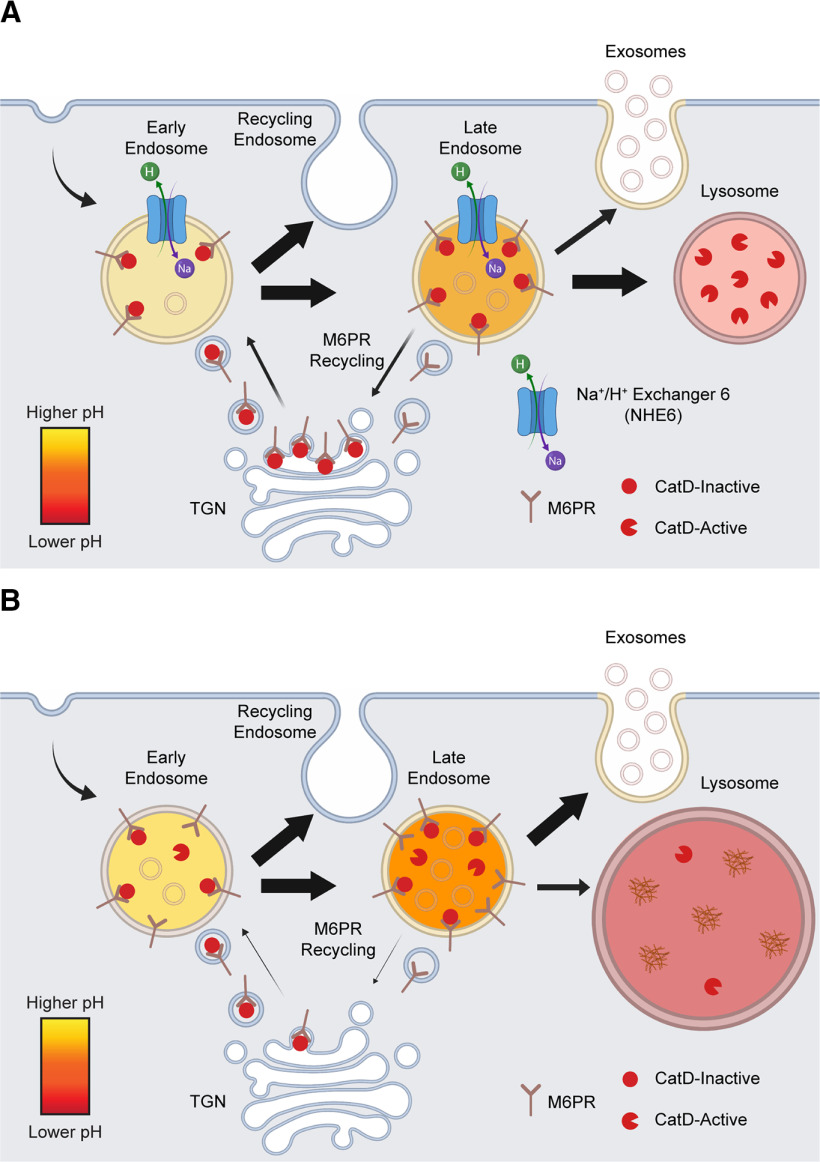
NHE6-null endolysosomal model in neurons. ***A***, Schematic representation of endosomal maturation and trafficking in WT neurons. Newly synthesized CatD enzymes are trafficked through the endocytic pathway by M6PRs until they reach the highly acidic lysosome lumen to assist in degradation, ensuring proper lysosome functioning. ***B***, Loss of NHE6 leads to overacidification of both the endosomal and lysosomal lumen that ultimately results in lysosome dysfunction. CatD becomes prematurely active in hyperacidified endosomal compartments yet is less likely to be trafficked and active in lysosomes, likely due in part to impaired endosome-lysosome fusion. Trafficking of M6PRs, which are responsible for delivering newly synthesized CatD to lysosomes, is also disrupted as they accumulate in endosomes and are unable to be transported back to the TGN. Endolysosomal trafficking is further altered as MVBs are more likely to fuse with the PM, resulting in enhanced exosome release.

Loss-of-function mutations of the endosomal protein NHE6 cause CS, an X-linked disorder characterized by severe neurodevelopmental as well as neurodegenerative pathology (Gilfillan et al., 2008; Garbern et al., 2010; Pescosolido et al., 2014). Loss of NHE6 has previously been shown to hyperacidify endosomal compartments and alter endosomal signaling in neurons (Ouyang et al., 2013; Kucharava et al., 2020). However, we currently lack a comprehensive understanding of how the endolysosomal pathway is affected in NHE6-null neurons. In this study, we found that loss of NHE6 in primary hippocampal neurons leads to worsening lysosome functioning with days in culture, likely because of impaired endosome maturation and trafficking (Fig. 13). We present evidence of precocious activation of pH-dependent proteases, such as CatD, in endosomes, with reduced delivery of CatD to lysosomes because of reduced endosome-lysosome fusion. We also present evidence of accumulation of M6PRs in late endosomes, potentially reflecting defective retromer function. Coincident with these late endosome trafficking defects, we find enhanced fusion of late endosomes or MVBs with the PM and enhanced exosome release in NHE6-null neurons.

Neuropathological findings indicative of lysosome deficiency have been reported in a CS mouse model (Stromme et al., 2011; Sikora et al., 2016). These *in vivo* results are important as they strengthen the significance of our mechanistic studies here, indicating that our studies are not strictly attributable to the *in vitro* setting. In Stromme et al. (2011), NHE6-null mice exhibit features consistent with lysosomal storage diseases, such as pathologic accumulation of GM2 ganglioside and unesterified cholesterol in late endosomes and lysosomes that affect particular brain regions, including the hippocampus. In the current study, we directly measured lysosomal degradation of endocytosed cargo *in vitro*. NHE6-null neurons displayed significantly less overall DQ-BSA fluorescence, indicating reduced overall degradative capacity, consistent with lysosome deficiency. Furthermore, it is unlikely that this difference in BSA degradation is because of less BSA being internalized in NHE6-null neurons, as we observed equivalent endocytosis of fluorescent BSA. Importantly, we observed worsening lysosome function in NHE6-null neurons with time in culture, suggesting that these defects may be secondary to endosomal trafficking defects accumulated over time.

We examined specific lysosome enzymes, including CatD, a pH-dependent aspartic lysosomal hydrolase. CatD gene mutations have been identified in the lysosomal storage disease neuronal ceroid-lipofuscinosis subtype 10 (CLN10) (Siintola et al., 2006; Steinfeld et al., 2006). We found that loss of NHE6 leads to decreased mature, enzymatically active CatD both *in vitro* and *ex vivo* in NHE6-null tissue. NHE6-null neurons demonstrated overall decreased active CatD fluorescence and number of puncta, as measured by BODIPY-pepstatin A. A limitation to our imaging experiments is that, while this probe is routinely used to visualize active CatD, pepstatin A is an aspartic protease inhibitor (Marciniszyn et al., 1976). Therefore, we cannot exclude that other aspartic proteases (e.g., cathepsin E, BACE1, etc.) are also labeled. However, our *ex vivo* experiments further support our interpretation that loss of NHE6 leads to decreased active CatD specifically. We found decreased mature CatD protein levels in 8-week-old NHE6-null hippocampal tissue compared with WT male littermates by western blotting.

We also observed a unique endolysosomal distribution of active CatD in NHE6-null neurons, consistent with precocious activation of CatD in endosomes. Notably, acidification-dependent dissociation of M6PR-ligand complexes occurs at a lower pH (∼5.8) than other ligands, such as insulin, consistent with dissociation in late endosome compartments (Borden et al., 1990); therefore, it may be possible that some level of CatD may be mislocalized because of premature dissociations from M6PR. Furthermore, reduced endosome signaling, as observed previously (Ouyang et al., 2013; Kucharava et al., 2020), may be in part because of premature dissociation of ligand and/or enhanced protease degradation of ligand-receptor complexes within endosomes. Importantly and in contrast, we observe less colocalization of active CatD with lysosomal markers (e.g., dextran and LAMP1). Our findings that active CatD was less likely to colocalize with lysosome-associated markers in NHE6-null neurons were further corroborated by our analysis of LEF brain tissue. NHE6-null LEF samples contained significantly less protein levels of both the enzymatically inactive pro-form as well as the active cleaved-form. These results, along with reduced endosome-lysosome fusion, suggest that CatD is not properly trafficked to degradative lysosomes, which is a shared pathobiologic feature across some lysosomal storage disorders (Futerman and van Meer, 2004, 2012; Platt et al., 2018).

NHE6 plays a role in regulating the luminal pH of the endocytic pathway. Loss of NHE6 has been shown to overacidify the endosomal lumen in neurons (Ouyang et al., 2013). However, it was not known whether NHE6 was also involved in the regulation of lysosomal pH. We found that NHE6-null neurons had a significantly lower pH compared to WT male littermates. To our knowledge, the only other disease-associated finding of lysosome hyper-acidification was reported in patient fibroblasts with a dominant, gain-of-function mutation in the Cl^–^/H^+^ exchanger *CLCN7* (Nicoli et al., 2019). Interestingly, these patients exhibited overlapping neurological features with CS, including cerebellar atrophy. These lysosome hyperacidification findings are in contrast to a number of mutations associated with neurological disease that impair lysosome acidification, such as *PS1* and *CLN1* (Lee et al, 2010, 2015; Colacurcio and Nixon, 2016; Bagh et al., 2017). Our *in vitro* findings are suggestive of NHE6 contributing to lysosomal pH homeostasis. Interestingly, reduced lysosomal pH may occur as a result of proton diffusion from the endosome compartment. Prior literature indicates that NHE6 is not localized to lysosomes (Brett et al., 2002; Ohgaki et al., 2010).

Studies of impaired lysosome enzyme trafficking in NHE6-null neurons suggest impairment in M6PR-dependent trafficking. In our studies here, we find reduced CatD and β-NAG, both M6PR-dependent enzymes (von Figura and Hasilik, 1986; Ghosh et al., 2003), but not reduced acid phosphatase, a M6PR-independent enzyme (Braun et al., 1989; Peters et al., 1990), in lysosomes. In Stromme et al. (2011), they found reduced β-Hex in hippocampus, a third M6PR-dependent enzyme (Hasilik and Neufeld, 1980). We observed that the steady-state distribution of M6PRs in NHE6-null neurons was skewed favoring greater colocalization with endolysosome markers, with strongest colocalization in late endosome and less colocalization with the TGN, suggesting defective retrograde trafficking from endosomes to the TGN, possibly involving retromer function. A consequence of this perturbed trafficking of M6PRs is decreased replenishment of the endocytic pathway of lysosome enzymes. Since we found no differences in M6PR protein levels, it is unlikely that insufficient M6PR protein is being produced in NHE6-null mice. Future experiments may examine retromer defects in NHE6-null neurons more directly. Overall, our data are consistent with lysosome deficiency caused by impaired trafficking of M6PR-dependent enzymes to lysosomes in NHE6-null neurons.

Late endosome fusion with the lysosome is a crucial step in the delivery of (1) newly synthesized lysosomal proteins and (2) endocytosed cargo for degradation (Luzio et al., 2007). Our findings support the conclusion that endosome-lysosome fusion is hampered in NHE6-null neurons *in vitro*. These results are consistent with a recent study of the yeast NHE6 ortholog, Nhx1, which shows that Nhx1 regulates MVB fusion with vacuoles (i.e., lysosome equivalent in yeast) (Karim and Brett, 2018). Currently, the specific molecular mechanisms for this fusion defect are not known. This could involve dysfunction of critical regulators of endosome-lysosome fusion, such as the molecular fusion machinery (Ballabio and Bonifacino, 2020). Interestingly, our data on accumulation of M6PR in RAB7 late endosomes suggest a second defect in late endosome trafficking, namely, potentially involving retromer function. A unifying hypothesis might suggest that a defect in late endosome maturation may concurrently lead to these distinct defects in late endosome trafficking. Importantly, it is also unknown whether autophagosome-lysosome fusion is delayed in NHE6-null neurons. Taken together, our findings are consistent with loss of NHE6 impairing late endosome maturation, and specifically the ability of endosomes to fuse with lysosomes, which reflects important new mechanistic insight into disease pathophysiology.

Our data using a novel CD63-pHluorin construct (Verweij et al., 2018; Bebelman et al., 2020) provide further evidence of altered late endosome trafficking in NHE6-null mice. Specifically, NHE6-null neurons demonstrate enhanced MVB fusion with the PM along with CD63-associated exosome release. We observe a very low basal rate of CD63-associated exosome release in control neurons. Endolysosome dysfunction has been shown to enhance exosomal secretion (Strauss et al., 2010; Villarroya-Beltri et al., 2016; Gauthier et al., 2017) as well as proteins associated with neurodegenerative disorders (Alvarez-Erviti et al., 2011; Miranda et al., 2018). Cells with compromised lysosome function may increase exosome secretion as a protective mechanism to bypass lysosomes and release endosomal cargo extracellularly (Levy, 2017; Miranda et al., 2018). While loss of NHE6 led to an upregulation in MVB/late endosome fusion with the PM in neurons *in vitro*, there were no differences in lysosome fusion with the PM (i.e., lysosomal exocytosis).

In summary, our study provides insight into how endolysosome functioning is perturbed by the loss of NHE6, underlying the pathophysiology of CS. We show that loss of NHE6 impairs lysosome degradative function as well as disrupts trafficking of endosomes to lysosomes. Interestingly, we observed NHE6-null neurons exhibit impaired endosome-lysosome fusion while, simultaneously, enhanced release of MVB-derived exosomes. CS may exemplify lysosome deficiency secondary to defects in endosome maturation and trafficking, broadening the spectrum of lysosome-related neurologic disorders. In conclusion, these studies indicate that, in addition to playing a role in regulation in intra-endosomal and lysosome pH, loss of NHE6 has important impact on endosome maturation and trafficking.

## References

[B1] Alvarez-Erviti L, Seow Y, Schapira AH, Gardiner C, Sargent IL, Wood MJ, Cooper JM (2011) Lysosomal dysfunction increases exosome-mediated alpha-synuclein release and transmission. Neurobiol Dis 42:360–367. 10.1016/j.nbd.2011.01.029 21303699 PMC3107939

[B2] Andrews NW (2000) Regulated secretion of conventional lysosomes. Trends Cell Biol 10:316–321. 10.1016/s0962-8924(00)01794-3 10884683

[B3] Arighi CN, Hartnell LM, Aguilar RC, Haft CR, Bonifacino JS (2004) Role of the mammalian retromer in sorting of the cation-independent mannose 6-phosphate receptor. J Cell Biol 165:123–133. 10.1083/jcb.200312055 15078903 PMC2172094

[B4] Bagh MB, Peng S, Chandra G, Zhang Z, Singh SP, Pattabiraman N, Liu A, Mukherjee AB (2017) Misrouting of v-ATPase subunit V0a1 dysregulates lysosomal acidification in a neurodegenerative lysosomal storage disease model. Nat Commun 8:14612. 10.1038/ncomms14612 28266544 PMC5344305

[B5] Ballabio A, Bonifacino JS (2020) Lysosomes as dynamic regulators of cell and organismal homeostasis. Nat Rev Mol Cell Biol 21:101–118. 10.1038/s41580-019-0185-4 31768005

[B6] Bebelman MP, Bun P, Huveneers S, van Niel G, Pegtel DM, Verweij FJ (2020) Real-time imaging of multivesicular body-plasma membrane fusion to quantify exosome release from single cells. Nat Protoc 15:102–121. 10.1038/s41596-019-0245-4 31836866

[B7] Blott EJ, Griffiths GM (2002) Secretory lysosomes. Nat Rev Mol Cell Biol 3:122–131. 10.1038/nrm732 11836514

[B8] Bolte S, Cordelieres FP (2006) A guided tour into subcellular colocalization analysis in light microscopy. J Microsc 224:213–232. 10.1111/j.1365-2818.2006.01706.x 17210054

[B9] Bonsergent E, Lavieu G (2019) Content release of extracellular vesicles in a cell-free extract. FEBS Lett 593:1983–1992. 10.1002/1873-3468.13472 31175663

[B10] Bonsergent E, Grisard E, Buchrieser J, Schwartz O, Théry C, Lavieu G (2021) Quantitative characterization of extracellular vesicle uptake and content delivery within mammalian cells. Nat Commun 12:1864. 10.1038/s41467-021-22126-y 33767144 PMC7994380

[B11] Borden LA, Einstein R, Gabel CA, Maxfield FR (1990) Acidification-dependent dissociation of endocytosed insulin precedes that of endocytosed proteins bearing the mannose 6-phosphate recognition marker. J Biol Chem 265:8497–8504. 2160460

[B12] Boustany RM (2013) Lysosomal storage diseases: the horizon expands. Nat Rev Neurol 9:583–598. 10.1038/nrneurol.2013.163 23938739

[B13] Braulke T, Bonifacino JS (2009) Sorting of lysosomal proteins. Biochim Biophys Acta 1793:605–614. 10.1016/j.bbamcr.2008.10.016 19046998

[B14] Braun M, Waheed A, von Figura K (1989) Lysosomal acid phosphatase is transported to lysosomes via the cell surface. EMBO J 8:3633–3640. 2583113 10.1002/j.1460-2075.1989.tb08537.x PMC402045

[B15] Brett CL, Wei Y, Donowitz M, Rao R (2002) Human Na(+)/H(+) exchanger isoform 6 is found in recycling endosomes of cells, not in mitochondria. Am J Physiol Cell Physiol 282:C1031–C1041. 10.1152/ajpcell.00420.2001 11940519

[B16] Brown WJ, Goodhouse J, Farquhar MG (1986) Mannose-6-phosphate receptors for lysosomal enzymes cycle between the Golgi complex and endosomes. J Cell Biol 103:1235–1247. 10.1083/jcb.103.4.1235 2945825 PMC2114320

[B17] Canuel M, Korkidakis A, Konnyu K, Morales CR (2008) Sortilin mediates the lysosomal targeting of cathepsins D and H. Biochem Biophys Res Commun 373:292–297. 10.1016/j.bbrc.2008.06.021 18559255

[B18] Casey JR, Grinstein S, Orlowski J (2010) Sensors and regulators of intracellular pH. Nat Rev Mol Cell Biol 11:50–61. 10.1038/nrm2820 19997129

[B19] Chen CS, Chen WN, Zhou M, Arttamangkul S, Haugland RP (2000) Probing the cathepsin D using a BODIPY FL-pepstatin A: applications in fluorescence polarization and microscopy. J Biochem Biophys Methods 42:137–151. 10.1016/s0165-022x(00)00048-8 10737220

[B20] Cheng XT, Xie YX, Zhou B, Huang N, Farfel-Becker T, Sheng ZH (2018) Characterization of LAMP1-labeled nondegradative lysosomal and endocytic compartments in neurons. J Cell Biol 217:3127–3139. 10.1083/jcb.201711083 29695488 PMC6123004

[B21] Colacurcio DJ, Nixon RA (2016) Disorders of lysosomal acidification: the emerging role of v-ATPase in aging and neurodegenerative disease. Ageing Res Rev 32:75–88. 10.1016/j.arr.2016.05.004 27197071 PMC5112157

[B22] Colombo M, Raposo G, Thery C (2014) Biogenesis, secretion, and intercellular interactions of exosomes and other extracellular vesicles. Annu Rev Cell Dev Biol 30:255–289. 10.1146/annurev-cellbio-101512-122326 25288114

[B23] Craig AM, Banker G (1994) Neuronal polarity. Annu Rev Neurosci 17:267–310. 10.1146/annurev.ne.17.030194.001411 8210176

[B24] Cui Y, Carosi JM, Yang Z, Ariotti N, Kerr MC, Parton RG, Sargeant TJ, Teasdale RD (2019) Retromer has a selective function in cargo sorting via endosome transport carriers. J Cell Biol 218:615–631. 10.1083/jcb.201806153 30559172 PMC6363445

[B25] Dotti CG, Sullivan CA, Banker GA (1988) The establishment of polarity by hippocampal neurons in culture. J Neurosci 8:1454–1468. 10.1523/JNEUROSCI.08-04-01454.1988 3282038 PMC6569279

[B26] Fraldi A, Klein AD, Medina DL, Settembre C (2016) Brain disorders due to lysosomal dysfunction. Annu Rev Neurosci 39:277–295. 10.1146/annurev-neuro-070815-014031 27090953

[B27] Futerman AH, van Meer G (2004) The cell biology of lysosomal storage disorders. Nat Rev Mol Cell Biol 5:554–565. 10.1038/nrm1423 15232573

[B28] Garbern JY, Neumann M, Trojanowski JQ, Lee VM, Feldman G, Norris JW, Friez MJ, Schwartz CE, Stevenson R, Sima AA (2010) A mutation affecting the sodium/proton exchanger, SLC9A6, causes mental retardation with tau deposition. Brain 133:1391–1402. 10.1093/brain/awq071 20395263 PMC2859154

[B29] Gauthier SA, Perez-Gonzalez R, Sharma A, Huang FK, Alldred MJ, Pawlik M, Kaur G, Ginsberg SD, Neubert TA, Levy E (2017) Enhanced exosome secretion in Down syndrome brain: a protective mechanism to alleviate neuronal endosomal abnormalities. Acta Neuropathol Commun 5:65. 10.1186/s40478-017-0466-0 28851452 PMC5576289

[B30] Ghosh P, Dahms NM, Kornfeld S (2003) Mannose 6-phosphate receptors: new twists in the tale. Nat Rev Mol Cell Biol 4:202–212. 10.1038/nrm1050 12612639

[B31] Gilfillan GD, Selmer KK, Roxrud I, Smith R, Kyllerman M, Eiklid K, Kroken M, Mattingsdal M, Egeland T, Stenmark H, Sjøholm H, Server A, Samuelsson L, Christianson A, Tarpey P, Whibley A, Stratton MR, Futreal PA, Teague J, Edkins S, et al. (2008) SLC9A6 mutations cause X-linked mental retardation, microcephaly, epilepsy, and ataxia, a phenotype mimicking Angelman syndrome. Am J Hum Genet 82:1003–1010. 10.1016/j.ajhg.2008.01.013 18342287 PMC2427207

[B32] Gopalakrishnan MM, Grosch HW, Locatelli-Hoops S, Werth N, Smolenova E, Nettersheim M, Sandhoff K, Hasilik A (2004) Purified recombinant human prosaposin forms oligomers that bind procathepsin D and affect its autoactivation. Biochem J 383:507–515. 10.1042/BJ20040175 15255780 PMC1133744

[B33] Gowrishankar S, Yuan P, Wu Y, Schrag M, Paradise S, Grutzendler J, De Camilli P, Ferguson SM (2015) Massive accumulation of luminal protease-deficient axonal lysosomes at Alzheimer's disease amyloid plaques. Proc Natl Acad Sci USA 112:E3699–E3708. 10.1073/pnas.1510329112 26124111 PMC4507205

[B34] Hasilik A, Neufeld EF (1980) Biosynthesis of lysosomal enzymes in fibroblasts: phosphorylation of mannose residues. J Biol Chem 255:4946–4950. 6989822

[B35] Hirst J, Futter CE, Hopkins CR (1998) The kinetics of mannose 6-phosphate receptor trafficking in the endocytic pathway in HEp-2 cells: the receptor enters and rapidly leaves multivesicular endosomes without accumulating in a prelysosomal compartment. Mol Biol Cell 9:809–816. 10.1091/mbc.9.4.809 9529379 PMC25308

[B36] Huotari J, Helenius A (2011) Endosome maturation. EMBO J 30:3481–3500. 10.1038/emboj.2011.286 21878991 PMC3181477

[B37] Johnson DE, Ostrowski P, Jaumouille V, Grinstein S (2016) The position of lysosomes within the cell determines their luminal pH. J Cell Biol 212:677–692. 10.1083/jcb.201507112 26975849 PMC4792074

[B38] Karim MA, Brett CL (2018) The Na(+)(K(+))/H(+) exchanger Nhx1 controls multivesicular body-vacuolar lysosome fusion. Mol Biol Cell 29:317–325. 10.1091/mbc.E17-08-0496 29212874 PMC5996954

[B39] Kobayashi T, Stang E, Fang KS, de Moerloose P, Parton RG, Gruenberg J (1998) A lipid associated with the antiphospholipid syndrome regulates endosome structure and function. Nature 392:193–197. 10.1038/32440 9515966

[B40] Kucera A, Borg Distefano M, Berg-Larsen A, Skjeldal F, Repnik U, Bakke O, Progida C (2016) Spatiotemporal resolution of Rab9 and CI-MPR dynamics in the endocytic pathway. Traffic 17:211–229. 10.1111/tra.12357 26663757

[B41] Kucharava K, Brand Y, Albano G, Sekulic-Jablanovic M, Glutz A, Xian X, Herz J, Bodmer D, Fuster DG, Petkovic V (2020) Sodium-hydrogen exchanger 6 (NHE6) deficiency leads to hearing loss, via reduced endosomal signalling through the BDNF/Trk pathway. Sci Rep 10:3609. 10.1038/s41598-020-60262-5 32107410 PMC7046661

[B42] Kulkarni VV, Maday S (2018) Neuronal endosomes to lysosomes: a journey to the soma. J Cell Biol 217:2977–2979. 10.1083/jcb.201806139 30115668 PMC6123003

[B43] Laulagnier K, Schieber NL, Maritzen T, Haucke V, Parton RG, Gruenberg J (2011) Role of AP1 and Gadkin in the traffic of secretory endo-lysosomes. Mol Biol Cell 22:2068–2082. 10.1091/mbc.E11-03-0193 21525240 PMC3113771

[B44] Lee JH, Yu WH, Kumar A, Lee S, Mohan PS, Peterhoff CM, Wolfe DM, Martinez-Vicente M, Massey AC, Sovak G, Uchiyama Y, Westaway D, Cuervo AM, Nixon RA (2010) Lysosomal proteolysis and autophagy require presenilin 1 and are disrupted by Alzheimer-related PS1 mutations. Cell 141:1146–1158. 10.1016/j.cell.2010.05.008 20541250 PMC3647462

[B45] Lee JH, McBrayer MK, Wolfe DM, Haslett LJ, Kumar A, Sato Y, Lie PP, Mohan P, Coffey EE, Kompella U, Mitchell CH, Lloyd-Evans E, Nixon RA (2015) Presenilin 1 maintains lysosomal Ca(2+) homeostasis via TRPML1 by regulating vATPase-mediated lysosome acidification. Cell Rep 12:1430–1444. 10.1016/j.celrep.2015.07.050 26299959 PMC4558203

[B46] Levy E (2017) Exosomes in the diseased brain: first insights from in vivo studies. Front Neurosci 11:142. 10.3389/fnins.2017.00142 28386213 PMC5362612

[B47] Lie PPY, Nixon RA (2019) Lysosome trafficking and signaling in health and neurodegenerative diseases. Neurobiol Dis 122:94–105. 10.1016/j.nbd.2018.05.015 29859318 PMC6381838

[B48] Lin SX, Mallet WG, Huang AY, Maxfield FR (2004) Endocytosed cation-independent mannose 6-phosphate receptor traffics via the endocytic recycling compartment en route to the trans-Golgi network and a subpopulation of late endosomes. Mol Biol Cell 15:721–733. 10.1091/mbc.e03-07-0497 14595110 PMC329388

[B49] Lloyd-Evans E, Haslett LJ (2016) The lysosomal storage disease continuum with ageing-related neurodegenerative disease. Ageing Res Rev 32:104–121. 10.1016/j.arr.2016.07.005 27516378

[B50] Luzio JP, Pryor PR, Bright NA (2007) Lysosomes: fusion and function. Nat Rev Mol Cell Biol 8:622–632. 10.1038/nrm2217 17637737

[B51] Marciniszyn J Jr, Hartsuck JA, Tang J (1976) Mode of inhibition of acid proteases by pepstatin. J Biol Chem 251:7088–7094. 10.1016/S0021-9258(17)32945-9 993206

[B52] Mazzulli JR, Zunke F, Isacson O, Studer L, Krainc D (2016) alpha-Synuclein-induced lysosomal dysfunction occurs through disruptions in protein trafficking in human midbrain synucleinopathy models. Proc Natl Acad Sci USA 113:1931–1936. 10.1073/pnas.1520335113 26839413 PMC4763774

[B53] Medina DL, Fraldi A, Bouche V, Annunziata F, Mansueto G, Spampanato C, Puri C, Pignata A, Martina JA, Sardiello M, Palmieri M, Polishchuk R, Puertollano R, Ballabio A (2011) Transcriptional activation of lysosomal exocytosis promotes cellular clearance. Dev Cell 21:421–430. 10.1016/j.devcel.2011.07.016 21889421 PMC3173716

[B54] Mellman I, Fuchs R, Helenius A (1986) Acidification of the endocytic and exocytic pathways. Annu Rev Biochem 55:663–700. 10.1146/annurev.bi.55.070186.003311 2874766

[B55] Miranda AM, Lasiecka ZM, Xu Y, Neufeld J, Shahriar S, Simoes S, Chan RB, Oliveira TG, Small SA, Di Paolo G (2018) Neuronal lysosomal dysfunction releases exosomes harboring APP C-terminal fragments and unique lipid signatures. Nat Commun 9:291. 10.1038/s41467-017-02533-w 29348617 PMC5773483

[B56] Nicoli ER, Weston MR, Hackbarth M, Becerril A, Larson A, Zein WM, Baker PR, Burke JD, Dorward H, Davids M, Huang Y, Adams DR, Zerfas PM, Chen D, Markello TC, Toro C, Wood T, Elliott G, Vu M, Zheng W, et al. (2019) Lysosomal storage and albinism due to effects of a de novo CLCN7 variant on lysosomal acidification. Am J Hum Genet 104:1127–1138. 10.1016/j.ajhg.2019.04.008 31155284 PMC6562152

[B57] Ohgaki R, Matsushita M, Kanazawa H, Ogihara S, Hoekstra D, van Ijzendoorn SC (2010) The Na^+^/H^+^ exchanger NHE6 in the endosomal recycling system is involved in the development of apical bile canalicular surface domains in HepG2 cells. Mol Biol Cell 21:1293–1304. 10.1091/mbc.e09-09-0767 20130086 PMC2847532

[B58] Ouyang Q, Lizarraga SB, Schmidt M, Yang U, Gong J, Ellisor D, Kauer JA, Morrow EM (2013) Christianson syndrome protein NHE6 modulates TrkB endosomal signaling required for neuronal circuit development. Neuron 80:97–112. 10.1016/j.neuron.2013.07.043 24035762 PMC3830955

[B59] Ouyang Q, Joesch-Cohen L, Mishra S, Riaz HA, Schmidt M, Morrow EM (2019) Functional assessment in vivo of the mouse homolog of the human Ala-9-Ser NHE6 variant. eNeuro 6:ENEURO.0046-19.2019. 10.1523/ENEURO.0046-19.2019 PMC6893231 31676550

[B60] Parolini I, Federici C, Raggi C, Lugini L, Palleschi S, De Milito A, Coscia C, Iessi E, Logozzi M, Molinari A, Colone M, Tatti M, Sargiacomo M, Fais S (2009) Microenvironmental pH is a key factor for exosome traffic in tumor cells. J Biol Chem 284:34211–34222. 10.1074/jbc.M109.041152 19801663 PMC2797191

[B61] Pescosolido MF, Stein DM, Schmidt M, El Achkar CM, Sabbagh M, Rogg JM, Tantravahi U, McLean RL, Liu JS, Poduri A, Morrow EM (2014) Genetic and phenotypic diversity of NHE6 mutations in Christianson syndrome. Ann Neurol 76:581–593. 10.1002/ana.24225 25044251 PMC4304796

[B62] Peters C, Braun M, Weber B, Wendland M, Schmidt B, Pohlmann R, Waheed A, von Figura K (1990) Targeting of a lysosomal membrane protein: a tyrosine-containing endocytosis signal in the cytoplasmic tail of lysosomal acid phosphatase is necessary and sufficient for targeting to lysosomes. EMBO J 9:3497–3506. 2209556 10.1002/j.1460-2075.1990.tb07558.x PMC552098

[B63] Platt FM, Boland B, van der Spoel AC (2012) The cell biology of disease: lysosomal storage disorders: the cellular impact of lysosomal dysfunction. J Cell Biol 199:723–734. 10.1083/jcb.201208152 23185029 PMC3514785

[B64] Platt FM, d'Azzo A, Davidson BL, Neufeld EF, Tifft CJ (2018) Lysosomal storage diseases. Nat Rev Dis Primers 4:27. 10.1038/s41572-018-0025-4 30275469

[B65] Rodriguez A, Webster P, Ortego J, Andrews NW (1997) Lysosomes behave as Ca^2+^-regulated exocytic vesicles in fibroblasts and epithelial cells. J Cell Biol 137:93–104. 10.1083/jcb.137.1.93 9105039 PMC2139854

[B66] Scott CC, Vacca F, Gruenberg J (2014) Endosome maturation, transport and functions. Semin Cell Dev Biol 31:2–10. 10.1016/j.semcdb.2014.03.034 24709024

[B67] Seaman MN (2004) Cargo-selective endosomal sorting for retrieval to the Golgi requires retromer. J Cell Biol 165:111–122. 10.1083/jcb.200312034 15078902 PMC2172078

[B68] Sharma J, di Ronza A, Lotfi P, Sardiello M (2018) Lysosomes and brain health. Annu Rev Neurosci 41:255–276. 10.1146/annurev-neuro-080317-061804 29661037

[B69] Siintola E, Partanen S, Stromme P, Haapanen A, Haltia M, Maehlen J, Lehesjoki AE, Tyynela J (2006) Cathepsin D deficiency underlies congenital human neuronal ceroid-lipofuscinosis. Brain 129:1438–1445. 10.1093/brain/awl107 16670177

[B70] Sikora J, Leddy J, Gulinello M, Walkley SU (2016) X-linked Christianson syndrome: heterozygous female Slc9a6 knockout mice develop mosaic neuropathological changes and related behavioral abnormalities. Dis Model Mech 9:13–23. 10.1242/dmm.022780 26515654 PMC4728337

[B71] Steinfeld R, Reinhardt K, Schreiber K, Hillebrand M, Kraetzner R, Bruck W, Saftig P, Gartner J (2006) Cathepsin D deficiency is associated with a human neurodegenerative disorder. Am J Hum Genet 78:988–998. 10.1086/504159 16685649 PMC1474096

[B72] Strauss K, Goebel C, Runz H, Mobius W, Weiss S, Feussner I, Simons M, Schneider A (2010) Exosome secretion ameliorates lysosomal storage of cholesterol in Niemann-Pick type C disease. J Biol Chem 285:26279–26288. 10.1074/jbc.M110.134775 20554533 PMC2924046

[B73] Stromme P, Dobrenis K, Sillitoe RV, Gulinello M, Ali NF, Davidson C, Micsenyi MC, Stephney G, Ellevog L, Klungland A, Walkley SU (2011) X-linked Angelman-like syndrome caused by Slc9a6 knockout in mice exhibits evidence of endosomal-lysosomal dysfunction. Brain 134:3369–3383. 10.1093/brain/awr250 21964919 PMC3212719

[B74] van Weert AW, Dunn KW, Geuze HJ, Maxfield FR, Stoorvogel W (1995) Transport from late endosomes to lysosomes, but not sorting of integral membrane proteins in endosomes, depends on the vacuolar proton pump. J Cell Biol 130:821–834. 10.1083/jcb.130.4.821 7642700 PMC2199957

[B75] Vazquez CL, Colombo MI (2009) Assays to assess autophagy induction and fusion of autophagic vacuoles with a degradative compartment, using monodansylcadaverine (MDC) and DQ-BSA. Methods Enzymol 452:85–95. 10.1016/S0076-6879(08)03606-9 19200877

[B76] Verweij FJ, Bebelman MP, Jimenez CR, Garcia-Vallejo JJ, Janssen H, Neefjes J, Knol JC, de Goeij-de Haas R, Piersma SR, Baglio SR, Verhage M, Middeldorp JM, Zomer A, van Rheenen J, Coppolino MG, Hurbain I, Raposo G, Smit MJ, Toonen RF, van Niel G, et al. (2018) Quantifying exosome secretion from single cells reveals a modulatory role for GPCR signaling. J Cell Biol 217:1129–1142. 10.1083/jcb.201703206 29339438 PMC5839777

[B77] Vidoni C, Follo C, Savino M, Melone MA, Isidoro C (2016) The role of cathepsin D in the pathogenesis of human neurodegenerative disorders. Med Res Rev 36:845–870. 10.1002/med.21394 27114232

[B78] Villarroya-Beltri C, Baixauli F, Mittelbrunn M, Fernández-Delgado I, Torralba D, Moreno-Gonzalo O, Baldanta S, Enrich C, Guerra S, Sánchez-Madrid F (2016) ISGylation controls exosome secretion by promoting lysosomal degradation of MVB proteins. Nat Commun 7:13588. 10.1038/ncomms13588 27882925 PMC5123068

[B79] von Figura K, Hasilik A (1986) Lysosomal enzymes and their receptors. Annu Rev Biochem 55:167–193. 10.1146/annurev.bi.55.070186.001123 2943218

[B80] Winckler B, Faundez V, Maday S, Cai Q, Guimas Almeida C, Zhang H (2018) The endolysosomal system and proteostasis: from development to degeneration. J Neurosci 38:9364–9374. 10.1523/JNEUROSCI.1665-18.2018 30381428 PMC6209849

[B81] Yap CC, Digilio L, McMahon LP, Garcia AD, Winckler B (2018) Degradation of dendritic cargos requires Rab7-dependent transport to somatic lysosomes. J Cell Biol 217:3141–3159. 10.1083/jcb.201711039 29907658 PMC6122995

[B82] Zaidi N, Maurer A, Nieke S, Kalbacher H (2008) Cathepsin D: a cellular roadmap. Biochem Biophys Res Commun 376:5–9. 10.1016/j.bbrc.2008.08.099 18762174

